# Systematic Human Learning and Generalization From a Brief Tutorial With Explanatory Feedback

**DOI:** 10.1162/opmi_a_00123

**Published:** 2024-03-01

**Authors:** Andrew J. Nam, James L. McClelland

**Affiliations:** Department of Psychology, Stanford University, Stanford, CA, USA

**Keywords:** systematicity, generalization, abstraction, explanation

## Abstract

We investigate human adults’ ability to learn an abstract reasoning task quickly and to generalize outside of the range of training examples. Using a task based on a solution strategy in Sudoku, we provide Sudoku-naive participants with a brief instructional tutorial with explanatory feedback using a narrow range of training examples. We find that most participants who master the task do so within 10 practice trials and generalize well to puzzles outside of the training range. We also find that most of those who master the task can describe a valid solution strategy, and such participants perform better on transfer puzzles than those whose strategy descriptions are vague or incomplete. Interestingly, fewer than half of our human participants were successful in acquiring a valid solution strategy, and this ability was associated with completion of high school algebra and geometry. We consider the implications of these findings for understanding human systematic reasoning, as well as the challenges these findings pose for building computational models that capture all aspects of our findings, and we point toward a role for learning from instructions and explanations to support rapid learning and generalization.

## INTRODUCTION

Humans can sometimes learn new things from one or a few examples and generalize what they have learned broadly beyond the range of examples they have seen (Ahn et al., 1992; Frank et al., 2009; Reber, 1969; Stuhlmüller et al., 2010). However, such rapid learning and generalization is far from ubiquitous. There are cases in which the time-course, outcome, and mechanism of learning depend on what is to be learned and the conditions under which learning occurs. As one example, a considerable body of evidence supports the view that humans can use explicit, rule-based learning in situations where stimuli can be grouped into different categories by a simple rule, but must rely on a slower, implicit learning system when incommensurate stimulus dimensions must be integrated so that such rules are difficult to apply (Ashby et al., 1998). Indeed, humans’ natural tendency to rely on rule-based learning can interfere with learning effectiveness when information integration is required (Vermaercke et al., 2014) and in other settings where simple explicit rules do not apply (Reber, 1976). In another line of work, task framing had a dramatic impact on human learning and generalization (Sternberg & McClelland, 2012). Human subjects had to learn to predict whether an outcome would occur after the presentation of a display containing one or two objects. Those who were given task framing in which objects were described as coming from categories with different causal powers relied on causal inference and category-based generalization, while those who were instructed simply to attend to the objects that appeared in the display relied on associative learning and similarity-based generalization.

In the present work, we investigate learning and generalization of a highly systematic problem solving strategy. We focus on learning a problem solving strategy of this kind because such strategies are relevant to understanding many advanced human cognitive abilities. Logical reasoning, mathematical problem solving, and computer programming all rely heavily on highly generalizable problem solving skills. Both philosophers and cognitive scientists (Fodor, 1975; Marcus, 2003) consider such skills essential characteristics of human reasoning abilities, yet many people struggle to acquire such reasoning skills. Learning more about how humans acquire them may therefore have both theoretical and practical implications. Furthermore, it would be very useful for an artificial learning system to be able to learn from a one or a few examples and generalize to the full range of possible unseen examples, but many contemporary AI systems that have been successful in other ways still struggle to acquire new information rapidly and to generalize in human like ways (Berglund et al., 2023; Lake et al., 2017). By understanding more about the process by which humans acquire such skills, we hope to come a step closer to understanding more about how people learn in this type of learning setting, and more about how to build models that capture these aspects of human learning abilities.

A central issue that we believe requires deeper investigation is the role of instructions and explanations in humans’ ability to recognize and utilize structure within a novel domain. When understanding a new conceptual structure such as a cultural practice or scientific procedure (Ahn et al., 1992), it has been observed that experimenter-provided explanations facilitate participants’ learning from a single example. Humans can also learn to play Atari games by reading instructions about the games (Tsividis et al., 2017), or by watching 2 minutes of expert play (Lake et al., 2017), perhaps using prior knowledge to infer their own explanations, a process known as explanation-based reasoning (DeJong & Mooney, 1986). It has also been argued that formal education encourages related forms of learning, including identifying relevant and irrelevant features when classifying novel items into categories, and that this enhances generalizable reasoning abilities (Cole et al., 1971; Scribner & Cole, 1973).

To explore these issues, we introduce a task we call the hidden single task, based on a solving technique of the same name in Sudoku. Sudoku is appealing as a domain in which to explore the general features of human systematic reasoning ability, since a solution technique, such as the hidden single technique as presented in our experiment, can be described in simple language without the need to appeal to technical concepts, making it potentially accessible to a wide range of human participants. Indeed, although Sudoku superficially uses numbers, it involves no numerical or arithmetic reasoning, and swapping numbers for English letters, Greek letters, or even symbols does not have any significant effect on correctness (Brophy & Hahn, 2014).

The hidden single technique requires the solver to use the digits already present in a grid and the principle of mutual exclusivity to deduce the content of a single designated empty cell. Our task is characterized by the same symmetries, group properties, and combinatorics that characterize Sudoku in general (Felgenhauer & Jarvis, 2006; Russell & Jarvis, 2006), allowing procedural transformations, such as re-assigning the roles of digits, shuffling rows and/or columns, and rotating or transposing the grid. This allows us to explore the process of learning within a controlled task subspace and to assess how well learning generalizes outside of the narrow range of examples used in the tutorial and in an initial practice phase of the experiment.

In our study, participants with no prior exposure to Sudoku completed a guided, interactive tutorial walking through the solution of a single puzzle, explaining how each step contributed to the solution and requiring the participant to identify constraints among digits in the grid that determined the correct solution to the puzzle. Participants then went through 25 practice puzzles sampled from a narrow range, and received explanatory feedback when they made errors. Following this, they received 64 test puzzles with systematic variation in puzzle features, allowing assessment of generalization beyond the range of examples experienced during practice. Finally, they completed a questionnaire about the strategies they used, their level of general education, and their coursework in mathematics.

The design of our study allows us to address several questions. First, how rapidly do successful learners acquire the solution strategy? Second, after successfully learning the skill, how well do they generalize to out-of-distribution samples? Third, how well can those who acquire their solution strategy describe the strategy they use, and does performance co-vary with the ability to give a valid description? Lastly, how universal is the ability to acquire this new problem solving skill and if the ability to acquire the skill is not universal, what factors co-vary with people’s ability to learn the skill quickly and generalize it broadly? Although we focus on a specific example skill, we believe the answers we obtain to these questions will be relevant to understanding human learning and generalization in a wide range of systematic problem solving domains, and will provide benchmarks and clues that can inform efforts to achieve human-like performance in such domains in artificial systems.

Our results provide evidence for the following:

- Many participants successfully acquired the hidden single strategy, but many others did not. One third of our participants (those we call *solvers*) showed clear evidence of learning the full solution strategy from the tutorial and practice phase, while most of the remaining participants acquired a less successful strategy that yielded the correct solution only 50% of the time.
- Self-reported education, particularly education in high-school algebra and geometry, was associated with successful learning. Successful learning often occurred rapidly, with 50% of solvers demonstrating consistently high accuracy starting from the 3rd trial and 90% by the 10th.
- Solvers successfully generalized to puzzles outside of the training distribution, albeit with selective performance costs we will detail.
- Among the solvers, most articulated a description of a valid solution strategy, but some did not, and those that did tended to perform more accurately and generalize more effectively on transfer puzzles.

In the Discussion, we consider the implications of these findings for our understanding of the human ability to acquire systematic reasoning skills and for efforts to capture human learning abilities in machines.

## MATERIALS AND METHODS

### Design and Participants

After extensive pilot testing and refinement of our experimental protocol, we preregistered the design and some of the key analyses of the results of our experiment (see the preregistration document at https://osf.io/smf4b/). Both the pilot and final study were conducted using Amazon Mechanical Turk. Participants were required to be US nationals.

Several features of our design revolved around the goal of characterizing generalization performance of participants who successfully acquired a new skill through our explanatory tutorial and practice with a narrow range of problem variations. We used a stringent criterion of learning success (described below), and sought to obtain a large enough sample of participants who met this criterion to allow reliable detection of small but potentially meaningful accuracy differences between control and generalization test problems within the first few test trials after the practice phase, using findings from our pilot studies to guide our choice of sample sizes to allow replication of suspected effects. To this end, we recruited new batches of participants until we had at least 75 that met our criterion for solving the task by the end of the 25 practice trials. Participants received a base payment for completing the study plus a bonus for each puzzle solved on the first attempt (median total payment $9.93 in 47.3 minutes). Participants were not notified of the purpose of the experiment.

### Task Description

**Figure 1.**
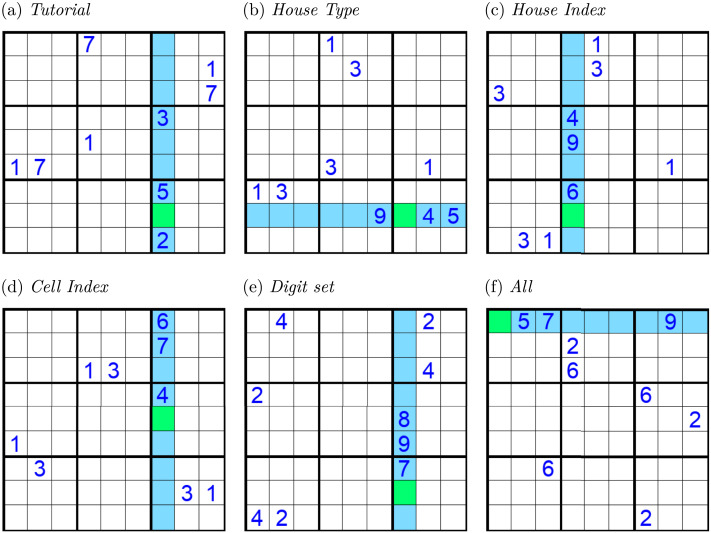
Examples of hidden single puzzles. Puzzles were generated using the participant’s individualized, random assignment of the training house type, house index, cell index, and digit set. (A) The full hidden single puzzle configuration as the participant might see it part way through the tutorial, conforming to the house type, house index, cell index, and containing the target (1) and the distractor (7) digits, both drawn from the training digit set. Examples used in the practice phase and control examples from the test phase would all use the same house type, house index, cell index, and training digit set, in this case the digits (1, 3, 5, 7). (B–F) Examples of non-control puzzles used in the test phase containing changes in features from the puzzles used in the tutorial and practice phase. (B–E) Puzzles with exactly one changed feature. (F) Puzzle with all four features changed.

The hidden single puzzles (Figure 1) follow the same constraints as Sudoku: in a valid solution, each row, column, and 3 × 3 box must contain exactly one instance of each number from 1 to 9. However, the hidden single puzzles are simplified so that there is a single *target* digit that must go into a green *goal* cell and not any other cell in the blue-highlighted *target house*. Puzzles were procedurally generated to have controlled variations while also maintaining standardized difficulty (see SI Section 1). Puzzles always contained 5 different digits called *hints*, one of which is the target and another of which is the *distractor*. The puzzles also always contained 3 unfilled cells in the target house within the same 3 × 3 box, which we refer to as the *box-constraint box*. The target and distractor digits each had 3 instances arranged in the grid in a way that prevented participants from performing reliably based on perceptually obvious heuristics such as counting the number of instances of a digit. There was always one target and one distractor constraining all three cells in the target house in the box-constraint box. The other three hints, called *in-house* digits, each occurred once in the highlighted house. The remaining four of nine digits, called *absent* digits, did not appear in the grid.

There are four experimentally controlled variable features of the puzzles. The *house type* is the type of house (row or column) to apply the hidden single technique to, indicated by whether it is a row or column that is highlighted in blue. The *house index* is the house to apply the hidden single technique to, also indicated by the blue highlighting. The *cell index* indicates which cell to solve for within a house, indicated by the cell highlighted in green. Lastly, the *digit set* is the set of 4 digits from which the target and distractor digits are drawn. The digits used in each puzzle are determined by first selecting a digit set, then assigning a number from the set as the target digit and a different number from the same set as the distractor digit. The three in-house digits are then randomly selected from the remaining 7 digits.

### Tutorial

**Figure 2.**
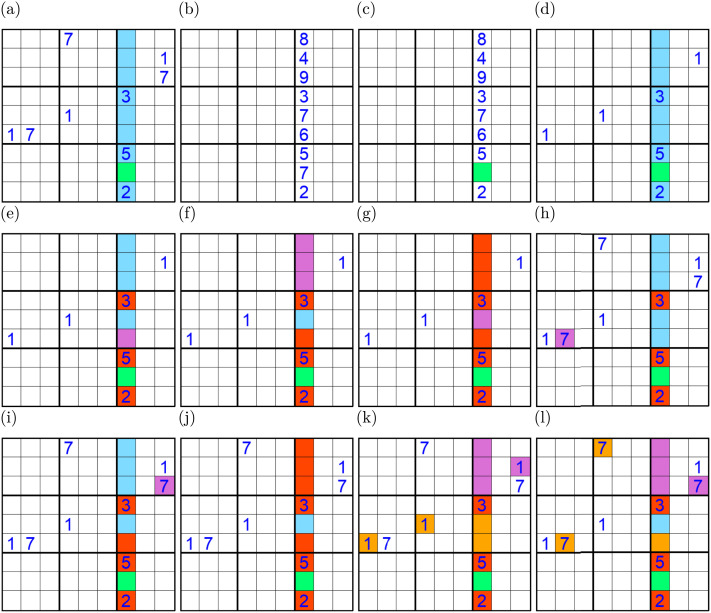
(A) The tutorial puzzle randomly generated for one participant. (B) The contradiction exercise. The goal is to select the two cells containing 7s. (C) The Full House exercise. The goal is to fill in the green cell with 1. (D) The tutorial puzzle with distractors removed. The goal is to enter 1 in the green cell. (E–G) Positive example tasks. The goal is to select the 1s that intersect with the purple cells. (H–J) Negative example tasks. The goal is to select the blue cells that intersect with the purple 7s. (K–L) Summary grids for both positive and negative examples.

At the start of the experiment, each participant was randomly assigned a specific house type (row or column), house and cell indices (both between 1 and 9), and a training set of four digits (all between 1 and 9) to be used in the tutorial puzzles, practice phase puzzles, and the control conditions in the test phase. For each participant, a *transfer* set of four digits was then selected from the 5 digits remaining. The experiment then began with a tutorial based on one hidden single puzzle randomly generated for each participant using the assigned puzzle features (Figure 2A shows an example). The restriction to a single puzzle was deliberate, as we wanted to later evaluate the participant’s ability to generalize to out-of-distribution transfer puzzles. In the example in Figure 1, the house type is column, the house index is 7 and the cell index is 8, and the target digit 1 and distractor digit 7 are chosen from the training digit set which also contains two other digits.

The tutorial began with a one-sentence description of Sudoku as “a puzzle with a 9 × 9 grid of numbers where each row, column, and 3 × 3 box must contain exactly one of each number from 1 to 9”. Beyond this initial statement, we took as much care as we could to avoid terms referring to variables and roles. Instead, we referred to specific digits by name and used different colors to highlight specific elements in the grid and refer to them, e.g., ‘the purple cell’ or the ‘blue column’. We intentionally avoided any abstract statements that described the strategy using general principles or rules and to avoid any indication that this pattern of reasoning could be applied beyond the exemplar puzzle. Throughout the tutorial, participants were required to enter responses that, if incorrect, resulted in an explanation of the reason the response was not correct, and participants were required to correct the error before proceeding. We next walk through most of the steps using the example shown in Figure 2.

On the first tutorial screen, below the one-sentence description of Sudoku stated above, the participant saw a grid with the target house in the assigned tutorial puzzle completely filled in (Figure 2B), but with two copies of the distractor digit 7, along with this text: “The column in the grid below does not contain every number between 1 and 9, but rather contains two copies of the digit 7, forming a contradiction. Select the two cells that create this contradiction.” After participants selected these cells, a new screen was presented (Figure 2C). Here, the goal cell was cleared and colored green, and the participant was asked to fill in the cell with the correct digit (this corresponds to a Sudoku solution technique known as the full house technique). Incorrect responses invoked feedback noting that the participant’s response digit was already present in the row or column.

Next, the tutorial walked the participant through a sequence of interactive screens demonstrating the solution steps to the participant’s specific puzzle, with each screen exemplifying an instance of a step in the general solution that could be applied to all of the puzzles used in the study. This was introduced with a screen that contained the tutorial puzzle, but with the distractor digits removed (Figure 2D). The screen included the text “In this section, we will focus on the green cell and solve for its value by looking along the blue column. Since the blue column must contain exactly one 1, let’s see if we can determine if the only place a 1 can go is in the green cell”. On the next screen, (Figure 2E), the participant was told “Obviously, a 1 cannot go in any of the red cells because they already contain numbers. Looking at the purple cell, we can see that there is a 1 in its row. This means we can eliminate 1 as a possible candidate for the purple cell”. The participant was then asked to click on the instance of the target digit that constrains a purple cell (here the correct answer would be the 1 in the left-most column). On the next screen, (Figure 2F) the previously purple cell was colored red, and the empty cells in the 3 × 3 box containing the target digit were colored purple. The participant was told “We’ve now successfully eliminated four possible cells that could contain a 1, now highlighted in red. Let’s now consider the 3 × 3 box containing a 1 and the three purple cells. There can only be a single 1 in the box, so the existing 1 prevents the three purple cells from containing a 1.” The participant was then asked to click on the 1 that was preventing the purple cells from being a 1. This was followed by a screen where the last empty cell other than the green target cell was highlighted in purple (Figure 2G), and the text “We have eliminated 7 cells (now highlighted in red) as possible candidates for 1 in the column containing the green cell, which means we now have only one cell to eliminate before we can definitely conclude that the green cell is a 1. Select the 1 that is preventing the purple cell from being a 1.” The next screen then said “We have successfully eliminated every cell in the blue column except for the green cell as potential candidates for 1. Fill in the green cell with the correct digit to solve this puzzle.” Following this, the 3 instances of the distractor digit (in our case, 7), were added and the participant was walked through a similar set of exercises providing an example of a digit that cannot be placed in the target cell because there was nothing preventing it from going in one of the blue cells (nothing prevents a 7 from going in the blue cell in the 5th row of the highlighted column in Figure 2). In this phase of the tutorial, participants were asked to identify the cells constrained by the instances of the distractor digit. For example, on a screen like the one in Figure 2H, they would need to select the blue cell in row six, which would then be colored red on the next screen (Figure 2I). This provided an alternative method of approaching the puzzle, starting with the clues to eliminate blue cells instead of checking if a blue cell could be eliminated as a possible location for a digit.

After a screen calling the participant’s attention to the cell that can contain a 7 (the remaining blue cell in Figure 2J), participants were shown color-coded summary grids for both the target and distractor side-by-side (Figure 2K and 2L) indicating the constraints on the grid and showing how all blue cells could be eliminated for the target but not for the distractor.

### Practice Phase

Following the tutorial, participants were given 25 puzzles to help reinforce what they learned during in the tutorial. All puzzles in the practice phase shared the same house type, goal cell position, and digit set as the tutorial, only varying in the hint locations and the choices of specific digits to serve in the various hint roles, subject to the constraints imposed by the digit set. However, the participants were not informed of the relationship between the puzzle used in the tutorial and the puzzles in this phase. Participants were allowed unlimited attempts and time to solve each puzzle with the goal of giving them the best chance of mastering the hidden single technique.

**Figure 3.**
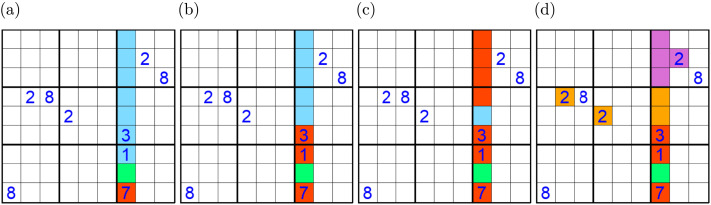
Sample feedback sequences during practice phase. (A) Feedback for submitting 7, an in-house response. (B) Feedback for submitting 4, an absent response. (C) Feedback for submitting 8, a distractor response. (D) Feedback for a correcting a previously incorrect response.

During this phase, all incorrect attempts produced detailed explanations specific to the puzzle and given response, referring to the particular digits and hints for why the response was incorrect. Figure 3 shows examples of the visual feedback participants would receive during the practice phase with the following accompanying text:

(a)  In-house feedback: 7 cannot be at the^1^ because 7 already exists in the same column in the red cell.
(b)  Absent feedback: It is not certain that 4 must be at the green cell because 4 may potentially be in a blue cell. The red cells cannot be 4 because they already contain digits.
(c)  Distractor feedback: It is not certain that 8 must be at the green cell because 8 may potentially be in the blue cell. Note that neither the green cell nor the blue cell share the same row, column, or box with a 8.
(d)  Target feedback (only after correcting an incorrect response): 2 is correct! We can be certain that the green cell must contain 2 because no other cell in its column can be a 2. The red cells cannot be 2 because they already contain digits. The empty purple cells cannot be 2 because they share the same box with a 2. The empty orange cells cannot be 2 because they share rows with other 2s.

Once the participant corrected an incorrect response, the participant was shown the source of the constraining hint for the eight non-goal cells in the target house, colored by the type of constraint (Figure 3D). Participants were given unlimited time to consider this display, clicking ‘Next’ when ready to proceed. No feedback was given for correct first responses except that they were correct.

### Test Phase

After the practice phase, participants were given 64 puzzles with systematic variations from the puzzles encountered thus far to evaluate transfer ability. We defined four conditions based on whether or not a puzzle differed from the tutorial and practice puzzles along the four features (digit set, house type, house index, cell index), yielding 16 combinations of possible puzzle conditions (including control puzzles which share all features with the tutorial and practice phase puzzles). The trials were internally (not revealed to participants) arranged into 8 sets of 8, where every set contained all 8 combinations of house type, house index, and cell index conditions. Within each set of 8 puzzles, 4 puzzles contained a change in the digit set. Every two sets of 8 puzzles contained all 16 combinations of house type, house index, cell index, and digit set conditions. These 8 combinations were arranged in a balanced Latin square such that each combination occurred once in each set, once in each position within a set, and 4 times before every other combination and 4 times after. During this phase, participants were allowed a single attempt and a 2-minute time limit for each puzzle. After submitting their responses, participants only received feedback on whether the response was correct or incorrect.

## RESULTS

### Separating Sudoku-Naive Participants Into Solvers and Non-Solvers

As a first step toward investigating rapid learning and generalization of the hidden single Sudoku strategy, we first identified a group of Sudoku-naive participants and then classified them into two groups based on whether they acquired a successful solution strategy from the tutorial and practice phases of our experiment. 1,985 people entered the study and after screening out 1,714 who demonstrated or attested to prior Sudoku ability or experience (See SI Section 1), we collected data from 271 apparently Sudoku-naive participants (age: *M* = 39, *SD* = 12; gender: 119 male, 149 female, 1 other, 2 no response). Of these, a subset who we refer to as *solvers*, acquired a successful solution strategy within the 25 puzzles of the practice phase according to our preregistered classification method, developed during pilot studies. Specifically, we fitted the following logistic regression (Equation 1) to predict the accuracy of the practice phase trials and classified participants as solvers if their predicted accuracy on the 25th trial exceeded a decision threshold of 0.8.

$$P\left(\text{correct}|\text{trial},\;\text{participant\_id}\right)∼\underset{\text{fixed}\;\text{effect}}{\log_{2}\;\text{trial}}+\underset{\text{random}\;\text{effects}}{\left(1+\log_{2}\;\text{trial}|\text{participant\_id}\right)}$$ (1)

**Figure 4.**
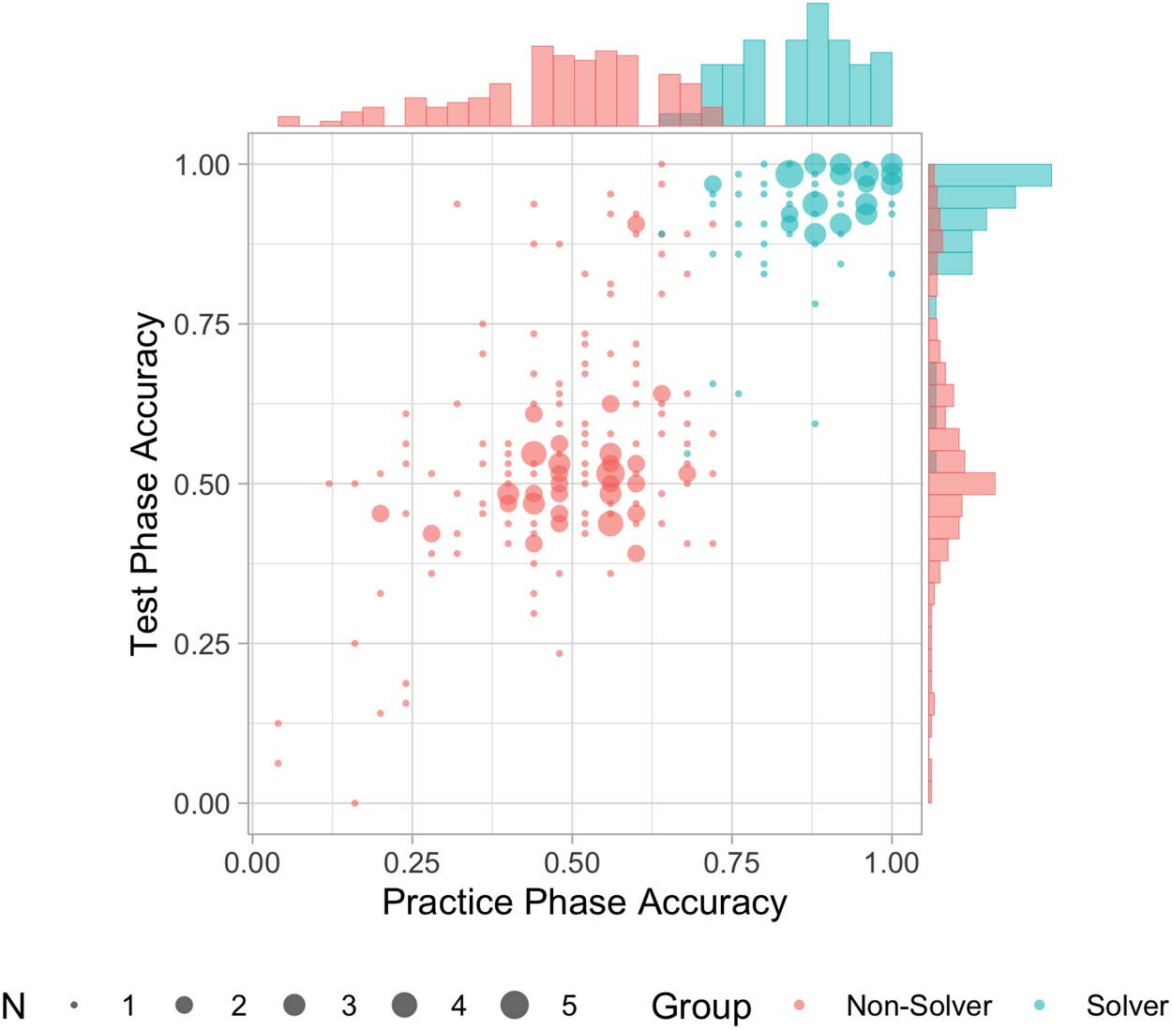
Overall accuracy during the practice and test phases. Each point represents *n* = 1 to 5 solvers or non-solvers with identical scores. Marginal histograms show the proportion of individuals in each group achieving each level of accuracy in each phase.

We note that a strategy of simply guessing between the target and distractor, the most common method used by non-solvers, would have less than 1% probability of solving 75% or more of the practice phase puzzles. Thus, few if any guessers are likely to have met this threshold. We identified 88 participants who met our criterion, leaving 183 participants whom we refer to as *non-solvers*. Figure 4 shows the overall accuracy of each participant in the experiment, color coded by solver status, plotting the participant’s accuracy in the test phase against their accuracy in the practice phase. The bulk of those classified as solvers solved puzzles with very high levels of accuracy in the test phase, while most non-solvers appeared to have adopted a strategy they continued to employ throughout the test phase, in which they chose between the target and the distractor with only a 50% chance of being correct. However, a subset of those classified as non-solvers achieved high levels of accuracy during the test phase, and some others may have adopted partially successful strategies (see SI Section 4). In what follows, we focus primarily on the performance of the solvers, contrasting their performance with that of non-solvers in certain cases.

### Dynamics of Strategy Acquisition

Since all puzzles used in our experiment share the same isomorphic structure, we classify responses to a puzzle into four categories based on the role of the digit given as the response: 1. *target*: the correct digit for the goal cell, which appears three times in each puzzle; 2. *distractor*: the incorrect, non-target digit that also appears three times in each puzzle; 3. *absent*: any digit that does not appear in the puzzle at all; and 4. *in-house*: any digit that already appears in the blue-highlighted house. Each puzzle contains one, one, four, and three digits in the target, distractor, absent and in-house classes, respectively. In the example puzzle in Figure 1A, these digits are {1}, {7}, {4, 6, 8, 9}, {2, 3, 5}.

The response categories also help define four different strategy classes based on which responses are expected to occur under each: 1. *uninformed guess* (UG): responses are completely unconstrained by the hints in the puzzle, selecting from any of the 9 digits with equal probability. 2. *avoid direct contradictions* (ADC): responses only avoid in-house digits, selecting from any of the remaining 6 digits with equal probability. 3. *prevalent digits* (PD): response are chosen by selecting one of the prevalent digits (ie the target or distractor) selecting between them with equal probability. 4. *successful* (S): the target digit is consistently selected.

Ignoring errors in strategy execution, the four strategy classes are expected to produce correct responses with probabilities 11.1%, 16.7%, 50%, and 100% respectively. Note that different specific strategies can produce responses consistent with each of the listed strategy classes. For example, a participant choosing randomly between the two prevalent digits, and a participant choosing between them on an arbitrary basis unrelated to the correctness of the response (e.g., always choosing the numerically larger of the two) would both be assigned to the PD strategy class. While other strategies and heuristics may be possible (see SI Section 4 basis A), these strategy classes account for most of the participants’ response profiles and are consistent with post-experiment survey responses (Section 4). Below we use the word ‘strategy’ to refer to a strategy class unless otherwise indicated.

Using these strategy definitions, we model the participants’ aggregate pattern of responses by assigning a weight to each strategy at each trial. First, combining the normative response probability for each strategy and how many digits belong to each category, we define a response emission matrix **R** where **R**_*i*,*j*_ = *P*(*r*_*j*_|*s*_*i*_), or the probability that a response in category *r*_*j*_ would be produced under strategy *s*_*i*_. However, participants may make errors, such as selecting a distractor digit even when using a successful strategy. We account for possible errors by allowing each strategy to occasionally fall back onto weaker strategies, but never stronger ones, represented by an error matrix **W** where **W**_*i*,*j*_ is the probability that strategy *s*_*i*_ will use the response probabilities of strategy *s*_*j*_. Next, we use a transition probability matrix **X** where **X**_*i*,*j*_ = *P*($s_{j}^{\left(t+1\right)}$|$s_{i}^{\left(t\right)}$), representing the average rate that participants transition from state *s*_*i*_ to state *s*_*j*_. Presumably, participants would use the best strategy available to them and not regress to a worse strategy with a lower expected accuracy, such as using the ADC strategy after having discovered the PD strategy. We explicitly adopt this assumption in our model by setting the transition probability from superior strategies to inferior strategies to 0. Finally, we represent the probability that a participant begins the practice phase with each strategy using the vector **a**.

We use these parameters to define an aggregate model that describes the behavior of a group of participants collectively:

$$P\left(r^{\left(t\right)}\right)={aX}^{t-1}WR$$ (2)

$$a=\left[a_{1}\;a_{2}\;a_{3}\;a_{4}\right]$$

$$X=\left[\begin{array}{cccc} x_{11} & x_{12} & x_{13} & x_{14}\\ 0 & x_{22} & x_{23} & x_{24}\\ 0 & 0 & x_{33} & x_{34}\\ 0 & 0 & 0 & 1 \end{array}\right]\;W=\left[\begin{array}{cccc} w_{11} & 0 & 0 & 0\\ w_{21} & w_{22} & 0 & 0\\ w_{31} & w_{32} & w_{33} & 0\\ w_{41} & w_{42} & w_{43} & w_{44} \end{array}\right]\;R=\left[\begin{array}{cccc} 3/9 & 4/9 & 1/9 & 1/9\\ 0 & 4/6 & 1/6 & 1/6\\ 0 & 0 & 1/2 & 1/2\\ 0 & 0 & 0 & 1 \end{array}\right]$$

where ***X***^*t*−1^ represents the transition matrix ***X*** repeatedly multiplied by itself *t* − 1 times in the Markov chain.

Since we expect solvers and non-solvers to have very different behaviors, we fit the model parameters separately for each group. We find the maximum likelihood estimates of each parameter by minimizing the negative log-likelihood using gradient descent with Adam (Kingma & Ba, 2014):

$$𝓛=-\sum_{p}\sum_{t=1}^{25}\;r_{p}^{\left(t\right)}\log\left({aX}^{t-1}WR\right)$$ (3)

where $r_{p}^{\left(t\right)}$ is the one-hot vector representation of participant *p*’s response on trial *t*.

The fitted parameters are shown below using subscripts *s* and *n* to indicate solvers and non-solvers respectively. For convenience, we also show **WR** which contains the probability of each response under each strategy including the error rate, although **R** itself is not fitted.

$$a_{s}=\left[\begin{array}{cccc} 0.085 & 0.241 & 0.310 & 0.365 \end{array}\right]\;X_{s}=\left[\begin{array}{cccc} 0.000 & 0.999 & 0.000 & 0.000\\ 0 & 0.106 & 0.892 & 0.001\\ 0 & 0 & 0.783 & 0.217\\ 0 & 0 & 0 & 1 \end{array}\right]$$

$$W_{s}=\left[\begin{array}{cccc} 1.000 & 0 & 0 & 0\\ 0.000 & 1.000 & 0 & 0\\ 0.000 & 0.054 & 0.946 & 0\\ 0.000 & 0.000 & 0.090 & 0.910 \end{array}\right]\;W_{s}R=\left[\begin{array}{cccc} 0.333 & 0.444 & 0.111 & 0.111\\ 0.000 & 0.667 & 0.167 & 0.167\\ 0.000 & 0.036 & 0.482 & 0.482\\ 0.000 & 0.000 & 0.045 & 0.955 \end{array}\right]$$

$$a_{n}=\left[\begin{array}{cccc} 0.109 & 0.397 & 0.463 & 0.032 \end{array}\right]\;X_{n}=\left[\begin{array}{cccc} 0.560 & 0.001 & 0.437 & 0.002\\ 0 & 0.770 & 0.229 & 0.001\\ 0 & 0 & 0.993 & 0.007\\ 0 & 0 & 0 & 1 \end{array}\right]$$

$$W_{n}=\left[\begin{array}{cccc} 1.000 & 0 & 0 & 0\\ 0.109 & 0.891 & 0 & 0\\ 0.024 & 0.038 & 0.939 & 0\\ 0.016 & 0.016 & 0.185 & 0.783 \end{array}\right]\;W_{n}R=\left[\begin{array}{cccc} 0.333 & 0.444 & 0.111 & 0.111\\ 0.036 & 0.642 & 0.161 & 0.161\\ 0.008 & 0.036 & 0.478 & 0.478\\ 0.005 & 0.018 & 0.097 & 0.880 \end{array}\right]$$

Assuming that participants make their decisions using exactly one of these strategies on each puzzle, we can model individual participants using a hidden Markov model (HMM) that assigns a single strategy at each trial. Although there are 4^25^ combinations of strategies, due to the monotonic improvement constraint, our model only spans 3,276 possible sequences of strategies. We use the aggregate parameters as priors for inferring the probability that a participant used a particular strategy on a given trial. For a given strategy path *π*, its prior probability is

$$P\left(\pi \right)=P\left(s^{\left(1\right)},\ldots ,s^{\left(25\right)}\right)=P\left(s^{\left(1\right)}\right)P\left(s^{\left(2\right)}|s^{\left(1\right)}\right)\ldots P\left(s^{\left(25\right)}|s^{\left(24\right)}\right)$$ (4)

$$\;=a_{s^{\left(1\right)}}x_{s^{\left(1\right)}s^{\left(2\right)}}\ldots x_{s^{\left(24\right)}}x_{s^{\left(25\right)}}$$ (5)

The likelihood of a participant’s sequence of responses under *π* is

$$P\left(r^{\left(1\right)},\ldots ,r^{\left(25\right)}|\pi \right)=P\left(r^{\left(1\right)}|s^{\left(1\right)}\right),\ldots ,P\left(r^{\left(25\right)}|s^{\left(25\right)}\right)$$ (6)

$$\;=r_{s^{\left(1\right)}r^{\left(1\right)}}\ldots r_{s^{\left(25\right)}r^{\left(25\right)}}$$ (7)

where *r*_*ij*_ is the entry in **R** on row *i* and column *j*. We then use Bayes’ rule to find the posterior probability of a strategy path given a participant’s responses

$$P\left(\pi |r^{\left(1\right)},\ldots ,r^{\left(25\right)}\right)=\frac{P\left(r^{\left(1\right)},\ldots ,r^{\left(25\right)}|\pi \right)P\left(\pi \right)}{\sum_{i=1}^{3276}P\left(r^{\left(1\right)},\ldots ,r^{\left(25\right)}|\pi_{i}\right)P\left(\pi_{i}\right)}$$ (8)

#### Solvers’ Strategy Dynamics.

**Figure 5.**
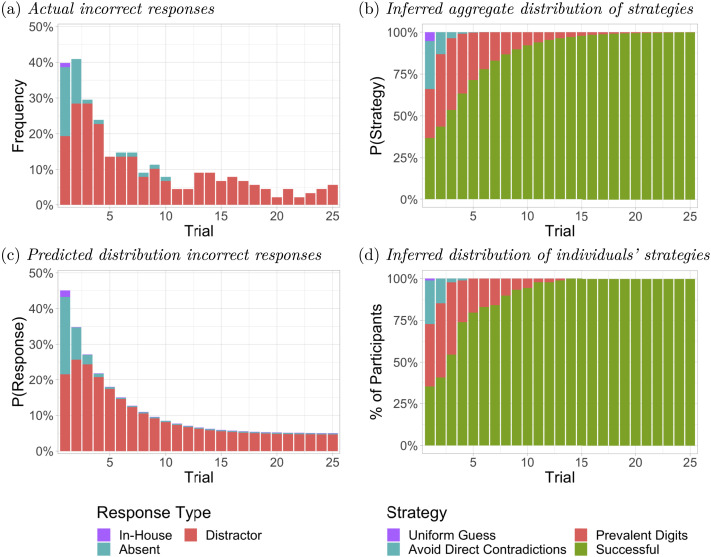
Response types and inferred strategies during the practice phase for participants identified as solvers. (A) Actual frequency of incorrect response types. (B) Inferred distribution of strategies by the aggregate model. (C) Predicted distribution of incorrect response types by the aggregate model. (D) Proportion of solvers inferred by individually fitted models to be using a strategy at least as effective as the strategy indicated.

The aggregate pattern of responses across trials in the practice phase (Figure 5A) suggest that participants in the solver group learned successful strategies well before the end of the practice phase (Figure 5B), demonstrating an impressive sample efficiency. As the figure shows, the inferred initial strategy distribution specifies that 36.8% of the responses were based on a successful strategy on the first practice trial, with 29.3%, 28.6% and 5.3% based on PD, ADC, and UG strategies respectively. Use of the UG and ADC strategies rapidly disappears, and the transition to a successful strategy is 92.1% complete by trial 10. The model’s corresponding expected response distribution, shown in Figure 5C, captures the main features of the pattern of the participants’ actual error responses shown in Figure 5A. The residual distractor responses shown are attributed to errors that occur with a probability of 4.5% under the successful strategy which is attributed to 50% of the responses on trials where successful solvers fall back on the PD strategy, with probability 0.09.

**Figure 6.**
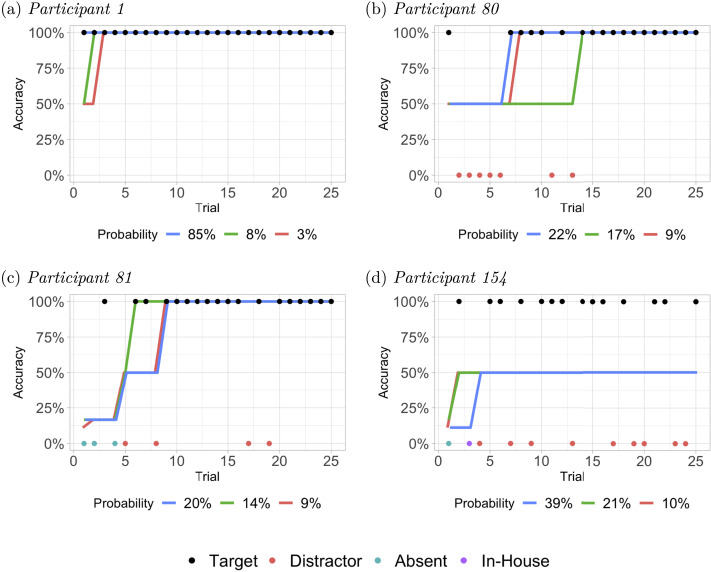
Responses of selected participants during the practice phase and their top 3 Viterbi paths. Panels (A), (B), and (C) show the profiles of solvers and (D) shows the profile of a non-solver. Strategy path probabilities were computed using individually fitted HMMs. Target (correct) responses are placed at accuracy of 100% whereas incorrect responses are placed at 0%. Viterbi path lines are placed horizontally at positions corresponding to the probability that responses based on the strategy will be correct, e.g., successful at 100% and prevalent digits at 50%, ignoring strategy execution errors.

Posterior probabilities of individual participants’ strategy paths reveal the different ways that they may have learned to solve the puzzles. Figure 6 shows the 3 most likely candidate strategy transition paths for four example participants, along with their response profiles. The uncertainty in these trajectories, particularly in the precise timing of the transition to a successful strategy as exhibited by participants in Figures 6B and 6C, arises because both target and distractor responses occur with equal probability under PD strategies, and distractor responses occasionally occur as errors under successful strategies. Even with an error-free participant (Figure 6A), the model assigns some probability to the possibility that some initial trials were lucky guesses under the PD strategy.

Given these uncertainties, we cannot know exactly when a particular solver transitioned to the successful strategy. By marginalizing over all possible paths, however, we can attain the probability that a participant used a particular strategy at each trial *P*(*s*_*t*_), which we can use to predict when the participant began to use different strategies. Taking the strategy with the highest posterior probability is problematic when the distribution does not strongly favor a particular strategy, for example if the posterior probabilities for all four strategies are close to 25%. However, what we can say with higher confidence is the probability that a participant used a strategy *s*_*t*_ that has an expected accuracy at least as high as strategy *s*: *P*(*s*_*t*_ ≥ *s*). Using this method, we can determine if a participant transitioned into strategy *s* at trial *t* by finding the first trial *t*_*x*_ when *P*(*s*_*t*_ ≥ *s*) > 50%:

$$t_{x}\left(s\right)=\underset{t}{\text{argmin}}\;P\left(s_{t}\geq s\right)$$ (9)

As shown in Figure 5D, we infer that 35.2% of the participants used a successful strategy from the first trial of the practice phase. By the same criterion, 54.5% of participants used a successful strategy by trial 3, 79.5% by trial 5, and 94.3% by trial 10.

#### Non-Solvers’ Strategy Dynamics.

**Figure 7.**
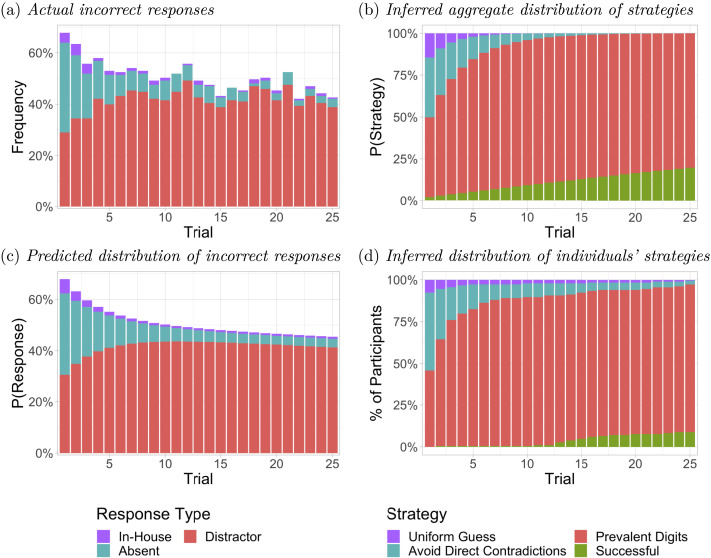
Response types and inferred strategies during the practice phase for participants identified as non-solver. (A) Actual frequency of incorrect response types. (B) Inferred distribution of strategies by the aggregate model. (C) Predicted distribution of incorrect response types by the aggregate model. (D) Proportion of non-solvers inferred by individually fitted models to be using a strategy at least as effective as the strategy indicated.

Although our main focus is on the solvers, we briefly present evidence of the strategies used by the non-solvers, based on fitting the same model used for the solvers to the data of the non-solvers (see Figure 7A). Considering first the actual incorrect responses of these participants, we see that they initially exceed 50%, and gradually approach about 50% correct by the end of the practice phase. The inferred distribution of strategies from the aggregate model indicates that on the very first trial of the practice phase, 14.5% of responses are attributed to uniform guessing, 35.7% to avoiding direct contradictions, 47.6% to choosing randomly between the prevalent digits, and only 2.2% to a successful solution strategy. Non-solvers tend to transition away from the UG and ADC strategies, with 80.3% of responses attributed to the PD strategy by the end of the practice phase, and smaller fractions attributable to successful (19.0%), ADC (0.1%) and UG (0.0%) strategies.

Even among responses attributable to a successful strategy, the HMM estimates an 88.0% actual correct response rate, significantly lower than the 95.5% of the solvers, with most of the remaining responses (9.7%) corresponding to the distractor. This higher rate of choices of the distractor digit make it much less certain when individuals who do transition to a successful strategy actually do so. That said, the cumulative estimates shown in Figure 7D can be used as an approximate guide to determining when these transitions may have occurred. By these estimates, most of these transitions occurred between trials 13 and 20, considerably later than nearly all of the estimated times for those in the solver group.

In summary, the bulk of the non-solvers appear to have adopted some variant of the PD strategy. A minority of participants classified as non-solvers may have acquired a successful strategy but failed to meet our preregistered solver criterion, in part because of a less reliable dependence on this strategy, and in part because they generally acquired the strategy later during the practice phase, or in some cases during the test phase.

### Solvers’ Generalization to Out-of-Distribution Puzzles

Returning to our focus on the 88 participants we identified as solvers at the end of the practice phase, we examine whether changing the digit set, house type, and goal cell position affected their accuracy and response times across the 64 test phase trials. Having observed potentially significant but short-lived effects of problem variations on accuracy and reaction time at the outset of the test phase on a pilot sample, we conducted preregistered analyses on performance on the first block of 16 trials separately from those in the remaining 3 blocks of 64 trials. The first 16 trials were chosen because that was when each participant saw all 16 combinations of changed and unchanged puzzle features. Overall, there was substantial transfer across all variations, as detailed below.

We used Bayesian mixed-effects models using BRMS (Bürkner, 2017) of the following preregistered form for our analyses with log-odds-ratio (logit) transformations for accuracy models and logarithmic transformations for response time (RT) models:

$$P\left(\text{correct}\right)∼\underset{\text{fixed}\;\text{effects}}{\text{feature}+\log_{2}\;\text{trial}}+\underset{\text{random}\;\text{effects}}{\left(1+\log_{2}\;\text{trial}|\text{participant\_id}\right)}$$ (10)

$$\log_{2}\;RT∼\underset{\text{fixed}\;\text{effects}}{\text{feature}+\log_{2}\;\text{trial}}+\underset{\text{random}\;\text{effects}}{\left(1+\log_{2}\;\text{trial}|\text{participant\_id}\right)}$$ (11)

We controlled for the practice effect using the log_2_ *trial* term as both fixed and random effects to account for individual differences. We report our analyses using the model specification as shown here and in the preregistration, but because overparameterization of random effects have been found to increase the chance of Type I errors (Matuschek et al., 2017; Oberauer, 2022), we also fitted models without the random slopes to check for possible false negatives and observed no meaningful differences in the model estimates or CIs (see SI Table 10 for side-by-side comparisons).

**Figure 8.**
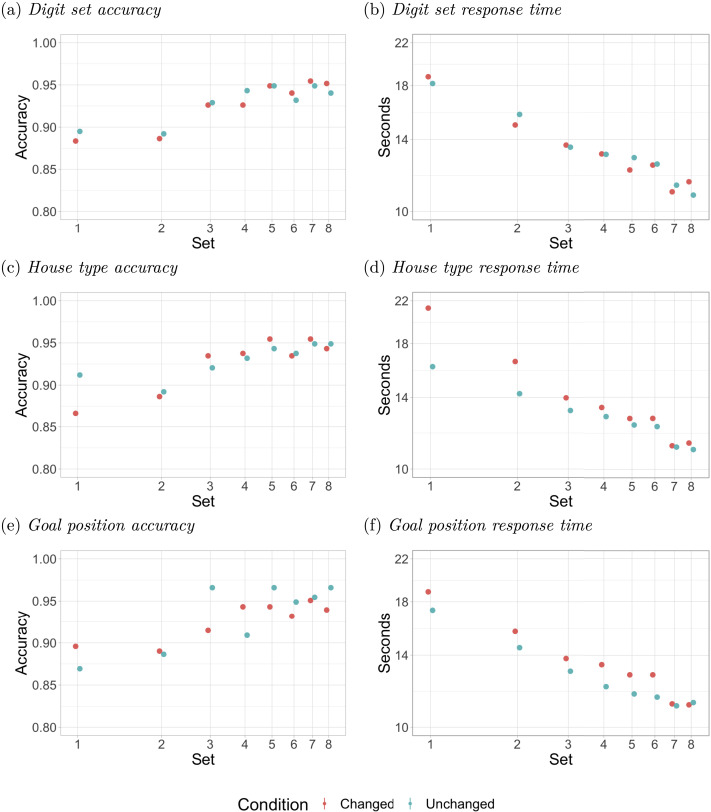
Average accuracy and response time in each set of 8 trials, e.g., set 1 contains trials 1–8 and set 2 contains 9 to 16. Note that the *y* axis for the response time plots and the *x* axis for all plots are in logarithmic scale. Only trials with correct responses included for response time plots.

We note that although house index and cell index were coded as two separate conditions for the experiment, and included as regressors in the preregistration, we chose to recode them as a single variable indicating change vs no change in the goal cell position for better interpretability (see SI Section 3). Table 1 shows the parameter estimates with 95% highest density credible intervals around the estimates with the parameter coefficients in logits for accuracy models and log_2_ (seconds) for response time models. Figure 8 shows average accuracy and response times during the test phase for each of the three conditions. We also report the Bayes factors in comparison to the null models, which we define as the regression formulas in Equations 10 and 11 without the *feature* parameter. Exact regression formulas, the full table of statistical measures, details of deviations from the preregistration, and regressions not reported in the main manuscript can be found in SI Sections 2 and 3.

**Table 1.** Test phase regression coefficient estimates.

Term	DV	Trials 1–16				Trials 17–64			
		Estimate	CI-L	CI-U	BF	Estimate	CI-L	CI-U	BF
DS	Acc.	−0.10	−0.46	0.26	0.50	0.01	−0.24	0.28	0.34
HT	Acc.	−0.30	−0.67	0.06	1.78	0.1	−0.18	0.37	0.52
GP	Acc.	0.16	−0.25	0.56	0.64	−0.31	−0.65	0.01	1.66
DS	RT	−0.02	−0.09	0.04	0.10	0.00	−0.03	0.03	0.04
HT	RT	0.33	0.26	0.38	1.94e20	0.05	0.02	0.09	9.40
GP	RT	0.11	0.04	0.19	9.24	0.09	0.05	0.13	2.97e3

DS: digit set; HT: house type; GP: goal cell position; CI-L and CI-H: lower and upper bounds of the 95% credible interval; BF: Bayes factor, where *BF* < 1 favors the null model and *BF* > 1 favors the augmented model. Accuracy (Acc.) coefficients are presented in logits and response time (RT) measure in coefficients are presented in log_2_ (seconds).

#### Digit Sets.

Solvers were able to transfer immediately when tested with target and distractor digits selected from a set never used in either of these roles during the tutorial or practice phases. Indeed, the effect of a change in the digit set, either for accuracy or response time, is negligible: all estimates are strikingly close to 0, falling well within the 95% CI, as shown in Table 1. Even in the first 16 trials where the transfer decrement would be greatest, the Bayes factors for the accuracy and RT models are 0.50 and 0.10, favoring the null model.

#### House Type.

Solvers were able to apply what they had learned after a switch in house type, albeit with a small initial reduction in accuracy and a substantial initial increase in response time. Although the 95% CI for the effect of a change in house type on accuracy includes 0, we note that about 90% of the probability mass is below 0 and an effect of similar size with 0 falling outside the 95% CI was obtained in pilot work reported in the preregistration of the current study. Thus, while this small accuracy decrement is likely to be a real effect, it is noteworthy that it is small and very short-lived: the effect was more prominent in the first half of the first block of 16 trials than in the second and is numerically reversed in trials 17–64. Response time increased by 0.32 log_2_ (s) in the first 16 trials, or roughly a 25% increase from an average of 19.59 seconds to 24.45 seconds. The effect on RT decreases rapidly, down to 0.05 log_2_ (s), or a 3.5% increase from 15.04 seconds to 15.58 seconds in the last 48 trials, and the effect appears to be gone by the end. There were no significant differences between the effect of changing from row to column and the effect of changing from column to row (see SI Section 2).

#### Goal Position.

A change in goal position did not significantly affect accuracy in the first 16 trials (0 fell well inside the CI) but produced a small decrease from 95% to 93% in the later 48 trials. For response times, we find main effects of 0.11 log_2_ (s) (1.08% increase) in the first 16 trials and of 0.09 log_2_ (s) (6.4% increase) in the last 48 trials. The 95% CI does not include 0 in either case.

In interpreting the effect of goal position, we note that, throughout the test phase, 25% of the puzzles used the same goal cell as the puzzles during the tutorial and practice phases, whereas when the goal position was changed, the goal cell would be any one of 16 cells with $\frac{1}{32}$ probability or of the remaining 64 cells with $\frac{1}{256}$ probability. Thus, the persistent effect of goal position might result from a justifiable bias in attention toward the most common goal cell location, producing a small cost when attention must be deployed to a less likely position. The Bayes factor of 2970 of the model for the last 48 trials compared to 9.24 of the first 16 indicates that the relative difference in RT increased during the test phase, which is consistent with the hypothesis that participants habituated to the modal goal cell. No such difference in relative likelihood applies either to the house type or the digit set variables, since both house types and both digit sets are used in the test problems with equal frequency.

### Explicitness of Acquired Strategies

Following the test phase, participants solved one last puzzle and were then asked to answer a free-response prompt: “Explain as clearly as possible the steps you went through to choose your answer. Please be as detailed as possible so that someone else could replicate your strategy by following your response.” For the following analyses, we sought to ensure that the verbal reports we considered were obtained from participants whose behavioral profiles were consistent with either successful or PD guessing strategies. Therefore, we screened out solvers who failed to maintain a high level of accuracy throughout the test phase and non-solvers whose pattern of responses was suggestive of strategies other than PD guessing (see SI Section 4). This left 84 participants in the group we call *persistent-solvers* and, coincidentally, exactly 84 participants in the group we call *PD-guessers*.

Two raters independently classified the responses of 168 participants into one of 9 categories. The first three categories represented three *valid* variations of the successful strategy that would yield the correct answer to the participant’s given puzzle, based on the rules of Sudoku and the specific constraints employed in constructing the puzzles used in the experiment. Three other categories represented *invalid* bases that would not reliably give the correct answer, such as randomly guessing between the two prevalent digits. Two categories were used for *uncertain* responses due to vague or unclear descriptions. A last category was used for *missing* or otherwise completely uninformative responses. Although all 168 responses were rated, we focus only on the responses of participants who correctly solved the final puzzle to avoid the possibility that differences between the ratings of responses by persistent solvers and PD guessers could be attributed to the rater’s awareness of the correctness of the solution, yielding 80 and 42 responses respectively for a total of 244 ratings between the two raters.

**Figure 9.**
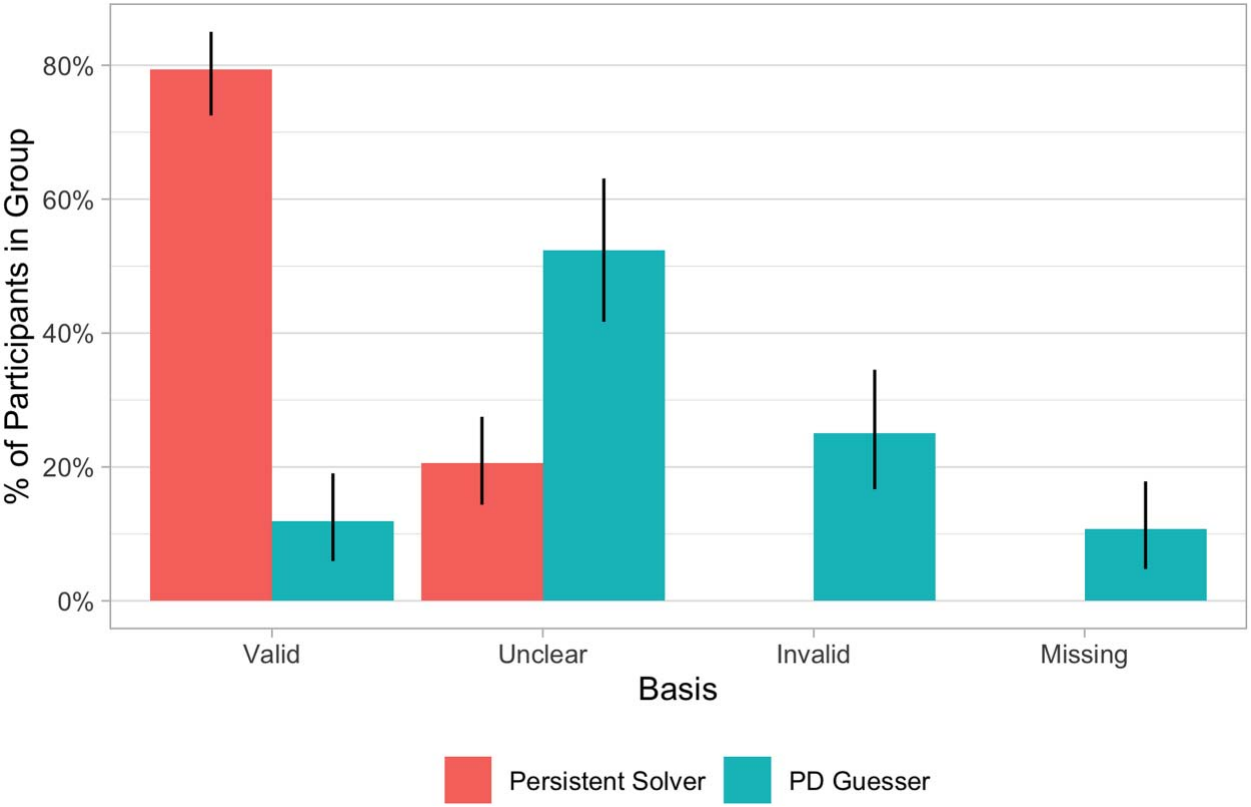
Ratings of self-reported strategies between persistent solvers and PD guessers.

As shown in Figure 9, 79% of persistent solvers’ strategy descriptions were rated as valid compared to 12% of PD guessers (*χ*^2^ = 99.12, *df* = 1, *p* < .001). Each bar represents the proportion of persistent solvers’ or PD guessers’ ratings (treating each rater’s rating of each of the participants as a separate rating). Using Cohen’s *κ* (Cohen, 1960; McHugh, 2012) to evaluate interrater agreement, we found that the two raters had substantial agreement across the 4 superordinate categories (*κ* = .80, CI = [.72, .88]) and across all 9 individual categories (*κ* = .63, CI = [.55, .72]), with most disagreements involving an uncertain rating from one of the raters.

#### Implicit vs. Explicit Reasoning.

Although some persistent solvers’ self-reported strategies were rated as vague or unclear, none were classified as unambiguously invalid. While some vague or unclear responses may be due to low effort from the participants, it is also possible that some participants actually reached consistently high accuracy without being able to put their strategies into words, i.e., that the strategies they acquired were implicit (Reber, 1989). The bulk of the solvers, however, did report valid strategies. Based on previous findings indicating that self-explanations often produce more robust understanding and better transfer to related problems (Chi et al., 1989, 1994), we hypothesized that solvers reporting strategies rated as valid might exhibit higher accuracy than the remaining solvers during the test phase, in which all but 1/16th of the puzzles fell outside of the range of puzzles used during the practice phase.

We tested this hypothesis by splitting the 80 persistent solvers that correctly solved the questionnaire puzzle into two groups: 60 *valid* solvers whose responses were classified as valid by both raters and 20 *unclear* solvers whose responses were classified as unclear by at least one rater. The valid solvers had an overall higher test phase accuracy than unclear solvers (95.2% vs. 92.1), despite having a lower practice phase accuracy (87.3% vs 91.8%). Controlling for the practice effect, valid solvers had higher odds of solving puzzles during the test phase compared to unclear solvers (0.593 logits, CI = [0.083, 1.125], corresponding to 1.81 higher odds), whereas unclear solvers had higher odds during the practice phase (0.582 logits, 95% CI = [0.023, 1.126], corresponding to 1.79 higher odds).

### Solver Status and Education

**Figure 10.**
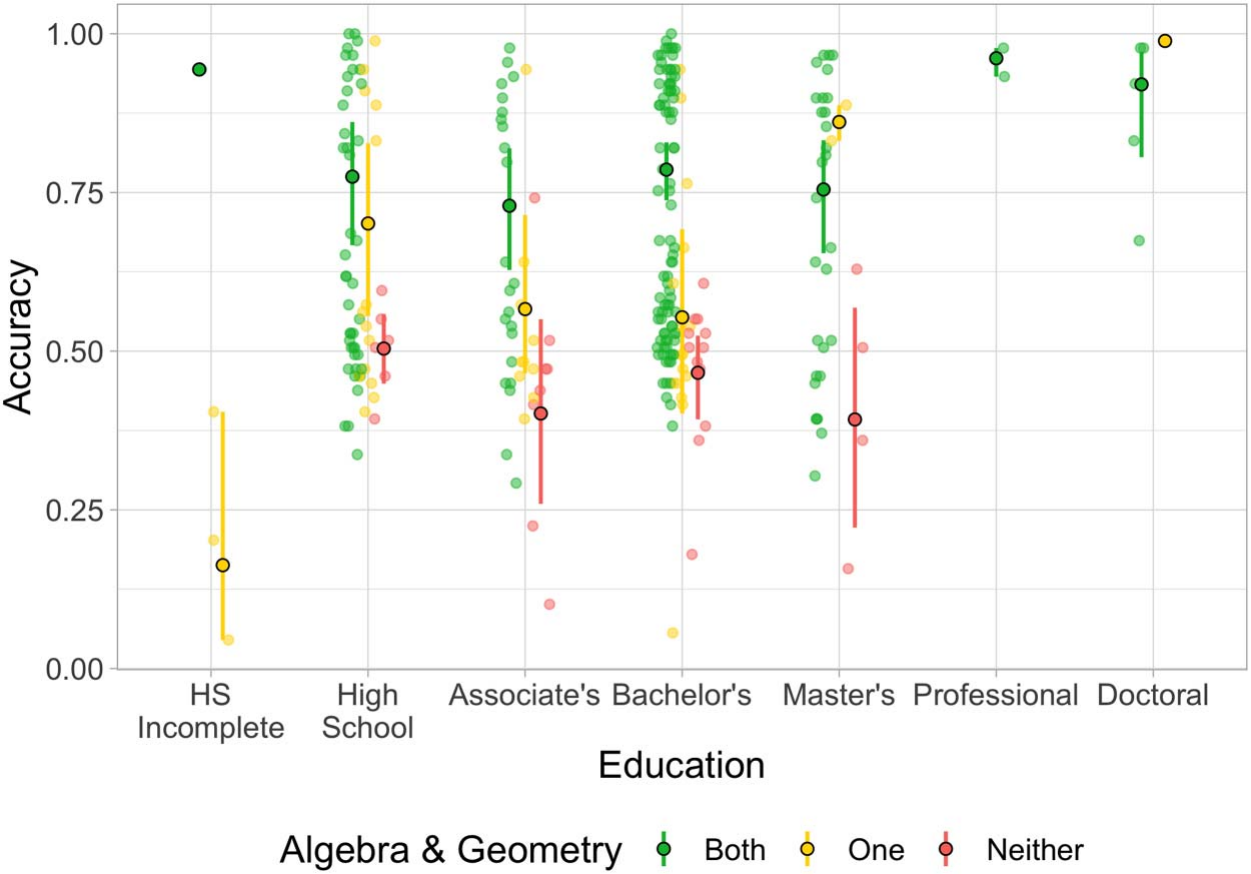
Overall accuracy across both the practice and test phases of the experiment by highest education. Color represents whether the participant has had formal education in both, one, or neither of algebra and geometry. Darker, bordered points represent group means and lighter points represent individual participants. All error bars show 95% bootstrapped confidence intervals (groups with *N* < 2 excluded). Means and intervals computed using log-odds of accuracy scores.

We next considered the relationship between education and participants’ ability to solve the puzzles (Figure 10). First, using self-report data on highest level of education pursued, we fitted a regression model to predict the total number of puzzles solved across both practice and test phases, finding that the number of years of education was a small but significant predictor of puzzles solved (*β* = 1.46, CI = [0.45, 2.45], *R*^2^ = 0.032). Next, we fitted a second regression using self-report data of various math courses taken and found that of the 9 different topics, only high-school algebra (*β* = 9.68, CI = [2.87, 16.78]) and high-school geometry (*β* = 9.81, CI = [3.78, 16.03]) were significant independent predictors. Notably, *none* of the 40 participants who reported having taken neither HS algebra nor HS geometry were solvers. In further analyses combining years of education and math courses, we found that education predicted a small amount of independent variance when combined with HS algebra and geometry (*R*^2^ = 0.168 with education, *R*^2^ = 0.152 without, Bayes factor = 11.517), but its significance is lost when considered together with all of the math course variables (*R*^2^ = 0.213 with education, *R*^2^ = 0.206 without, Bayes factor = 2.624). Conversely, including algebra and geometry in addition to years of education significantly increases *R*^2^ from 0.032 to 0.168 and the model fit by a Bayes factor of 2.802 × 10^10^.

## DISCUSSION

In this work, we explored how people learn to solve a systematic reasoning puzzle, how well they can extend their knowledge to handle new puzzles with previously unseen features, and how well they can describe their strategies. In light of the evidence presented in our work, we revisit the questions introduced at the beginning of this paper and their implications for our understanding of the human ability to acquire and generalize a systematic reasoning skill.

### Rapid Acquisition

How rapidly do successful learners acquire the solution strategy? We found that the majority of the successful learners began to reliably solve the hidden single puzzles within 10 trials, some even from the first trial beyond the initial tutorial. There was also a smaller group of participants categorized as non-solvers who might be called late solvers, in that they achieved high accuracies during the test phase, and some of these participants performed at or below 50% throughout the practice phase of the experiment. Thus, while the participants who learned the task generally did so from only a small number of examples, the distribution of learning times has a long tail, and the solution process in these individuals certainly deserves further investigation.

Focusing on those participants who met our criterion for successful learning within the practice phase, it is important to ask how they did so. In this context, it is relevant to remember that neither the tutorial nor the explanatory feedback described the hidden single technique in abstract terms, nor did the tutorial suggest how the procedure could be applied to other puzzles. One approach to understanding how successful participants found a solution so quickly would begin with the idea that they relied on abstract representations capturing the entities (digits, cells, and houses) in the puzzle and the crucial relations among them (e.g., the presence or absence of an instance of a digit in a house intersecting with a specific target cell). Such representations could have allowed them to construct a generalizable strategy from the examples described in the tutorial, with the further support from explanatory feedback after any errors they made within the first few practice trials. Within this proposal, they may have relied on prior knowledge and/or inductive biases that allowed them to construct and revise such representations quickly. As we will discuss below, this proposal is consistent with many other aspects of our findings.

### Systematic Generalization

After successfully learning the skill, how well do humans generalize to out-of-distribution samples? Strikingly, shifting from one set of digits to another produced no detectable change in accuracy or reaction time. Participants were slightly slowed by a switch of the target cell used during practice to a different cell, and such a switch could be associated with a switch to a different row or column from the one used during practice as well. As discussed in our results, since the target cell used in practice remained the most likely target cell throughout the test phase of the experiment, we interpret this small effect as reflecting a justifiable attentional bias favoring this cell rather than a learned strategy tied specifically to the target cell or to the specific row or column that cell was in. However, generalization from one house type to another produced a small initial drop in accuracy as well as a pronounced initial slowing of response time.

Considering first the generalization across digit sets, this is consistent with the idea that participants engaged with the digits in each puzzle as freely assignable to the various roles of digits within the puzzles, independently of what the identity of the digit happened to be. Indeed, our findings are consistent with the possibility that both solvers and non-solvers quickly came to appreciate that the two digits each occurring three times outside of the target house were the two they needed to consider as candidates, and that they could consider such a digit with equal facility whether or not they had encountered it in this role during practice. Similarly, we see our findings as consistent with the idea that participants could treat digits, rows or columns, and cells within columns interchangeably, with a slight bias to focus attention on the most frequently occurring target cell. This would be consistent with assigning cells and rows or columns to abstract roles, such as target cell, target row or or column, and (at least for solvers) intersecting row, column, or box.

The initial accuracy and speed decrement we observe when solvers switched from one house type to another suggests that they acquired a strategy during the practice phase that depended at least in part on whether their target house was a row or a column. Here, it is more difficult to determine whether or not participants strategies were initially abstract enough to treat rows and columns interchangeably at an underlying level. One possibility is that their underlying representations did involve such a high level of abstraction, but the switch in house type required an adjustment of the process required to map from the concrete display configuration into it. Another possibility is that participants were able to adapt a strategy specific to their initial house type onto a strategy that could be successfully applied to the orthogonal house type, perhaps relying on a structure-mapping or analogical reasoning process (Falkenhainer et al., 1989; Gick & Holyoak, 1980).

### Explicit Strategy Description

How well can those who acquire their solution strategy describe the strategy they use, and does performance co-vary with the ability to give a valid description? Most persistent solvers (those who met our solver criterion based on performance in the practice phase, and maintained at least an 80% level of performance during the test phase) provided descriptions of their solution strategies that could be identified as instances of specific valid strategies, possibly indicating that these individuals actually followed an explicit reasoning process consistent with their verbal descriptions. Interestingly, however, a quarter of the persistent solvers provided partially vague or incomplete descriptions, suggesting that they may have possessed at least vaguely cohesive explanations, even if they did not articulate them fully or clearly (Ahn et al., 1992; DeJong & Mooney, 1986). This finding suggests that people can acquire a form of knowledge that supports generalization without being able to describe their procedure in a written description—a form of implicit knowledge (Reber, 1989). Clarity of the strategy description was associated with better generalization and faster solution times during the test phase, where most of the puzzles required generalization in one way or another.

It is important to acknowledge that participants’ own descriptions of their strategies may not accurately reflect their solution processes. For instance, some solvers may not have fully reported explicit reasoning steps that they followed, or may have reasoned explicitly but with such proficiency that their strategy descriptions suffered from the expert blind spot (Nathan & Petrosino, 2003) due to weaker memory traces (Ericsson & Simon, 1980), resulting in being misclassified as unclear solvers. They also may have used multiple methods, which may have added to the difficulty of articulating a single strategy (Ericsson, 2017). Conversely, solvers could have learned and generalized without formulating explicit strategies and might have formulated such strategies upon being prompted, possibly even believing they had followed them all along (Nisbett & Wilson, 1977). Further research is needed to understand precisely how people engage in implicit and explicit abstract reasoning, and what differences arise as a consequence.

With these caveats having been noted, we would still suggest that it may be important to consider the possibility that solvers’ ability to provide accurate descriptions of their problem solving strategy may reflect a process that they engaged in as they worked through the tutorial and practice phases of our experiment. Our tutorial walked through only a single example, and the feedback we provided, while it indicated why an error response was wrong, did not provide participants with details of what to do to solve the given problem. Others have noted that students must often fill gaps or recall omitted argument steps when working through tutorial examples, and that those who do so are far more successful at learning and transfer than those who do not (Chi et al., 1989). Thus, to achieve success, successful learners may have constructed an explicit solution strategy for themselves, providing the basis for their ability to describe their solution strategy when prompted.

### Universality and Individual Differences

The final question we raised in the introduction asked: how universal is the ability to acquire the hidden single problem solving skill from our tutorial, and if the ability to acquire the skill is not universal, what factors co-vary with people’s ability to learn the skill quickly and generalize it broadly? We found that out of 271 participants, only 88, or roughly a third, of them could learn to solve the hidden single puzzles rapidly and reliably enough to meet our criterion for classifying them as solvers. We found evidence that mathematics education covaried with solver status: Specifically, we observed a reliable association between high-school mathematics education and solver status, and none of the participants lacking both high-school algebra and geometry backgrounds met our criteria for classification as solvers. We now consider how to think about this success rate and this relationship with mathematics education.

There is an important tradition that proposes that all humans rely on built-in systems that support rapid learning and systematic reasoning (Fodor & Pylyshyn, 1988; Lake et al., 2017), while others have suggested that that systematic reasoning ability of the kind exhibited by solvers is a consequence of formal schooling (Burger & Shaughnessy, 1986; Scribner & Cole, 1973; Vygotsky, 1934). Perhaps these ideas can be reconciled, at least in part, by thinking that the built in systems are implicit learning systems that support learning of near-universal human abilities such as natural language understanding. With simple enough statistics (such as strong sequential dependencies in syllable sequences) such implicit learning has been documented to occur with as few as 45 presentations of word-like sequences (Saffran et al., 1996) and sometimes generalizes to sequences that follow abstract rules without overlapping with the specific elements of the learning materials (Frank et al., 2009).

Our finding that only a subset of our Sudoku-naive participants were able to acquire the hidden single strategy indicates that, at least for tasks like those explored here, the relevant abilities are far from universal. What makes them different from those discussed above? As we have already discussed, the ability to acquire our hidden single strategy may depend on an ability to rely on a more explicit form of abstract relational reasoning, including understanding instructions and feedback and constructing for oneself an explicit strategy description. Interestingly, abstract relational reasoning underlies many tasks thought to tap into the key individual differences construct often called fluid intelligence and strongly associated with the factor-analytic construct called *g* (Gray & Thompson, 2004). If this ability is learnable, then formal schooling might well be relevant to it, as proposed by Vygotsky (1934) and others. However, there is considerable evidence that it is only when *multiple* relations need to be taken into account that a strong correlation with *g* emerges (Duncan et al., 2020; Waltz et al., 1999). In this context, it is worth noting that our hidden single task does require human subjects to consider multiple relationships—specifically the positional relationships between several instances of a candidate digit (the target or the distractor) and the empty non-target cells in the target house—in order to perform the task correctly, and there is evidence that human participants have more difficulty solving cells in Sudoku puzzles that require more relationships to be taken into account. A study by Lee et al. (2008) found that naive Sudoku players’ ability to solve for cells in full Sudoku puzzles depended strongly on the number of relations that had to be considered. The hidden single puzzles used in our experiment map onto a complexity rating of 4 in the measure proposed in Lee et al. (2008), corresponding to the highest complexity they attributed to any participant and higher than the complexity rating of 2.8 averaged over all of the participants.

A considerable body of evidence supports the view that the ability to solve problems requiring the consideration of multiple abstract relationships depends on brain mechanisms centered in the pre-frontal cortex that are associated with the ability to manage multiple demands, perform tasks relying on maintaining and manipulating items in working memory, and organizing behavior to confirm with specified task goals (Duncan et al., 2020; Gray & Thompson, 2004). While many have dreamed of finding ways to enhance the performance of these mechanisms, some studies find that efforts to do so do not transfer beyond the domains in which they are practiced (Ripp et al., 2022), suggesting that these mechanisms, themselves, are not directly subject to enhancement through experience. Yet there is clear evidence that intelligence test scores, including tests that depend on reasoning over multiple abstract relations, rose dramatically over the last century (Flynn, 1984), an effect many attribute to increases in the general level of education that occurred over the same period (Baker et al., 2015). It thus seems reasonably clear that complex relational reasoning is at least partially a consequence of education, and the same may be true of the ability to learn a new complex reasoning skill rapidly, as investigated in our experiment. In future work, we hope to explore the possibility that successful acquisition of our hidden single strategy depends on a synergistic, virtuous cycle between formal education and abstract reasoning ability, and that this synergy might play an important role in advanced human cognitive abilities. That is, individual differences in cognitive control, defined as the ability to manage multiple demands in service of specified goals, may be an important contributor to the human ability to engage in relational reasoning when multiple relationships are involved, and this in turn could contribute to human success in learning, using, and even inventing formal reasoning systems (McClelland, 2022).

In sum, we have found that a subset of our participants robustly acquired the hidden single strategy from our brief explanatory tutorial and practice with feedback, and that these participants were successful in generalizing to out-of-distribution puzzles. Most of these participants articulated valid strategies, and those who did performed better on the transfer test; and success in acquiring the hidden single strategy was associated with mathematics education. These findings are consistent with the idea that most successful participants engaged in structured, at least partially abstract, and often explicit reasoning and self-explanation, and with the idea that their ability to do so may depend on a synergistic interplay of cognitive control and formal education. With these ideas in consideration, we now turn our attention to their implications for modeling systematic and algorithmic reasoning in modern machine learning-based systems.

### Implications for Computational Models of Intelligence

Psychologists and computer scientists alike have long attempted to capture human-level intelligence through computational models. While numerous methods have been used to model intelligent behavior, such as rule-based systems (Newell & Simon, 1961) and probabilistic models (Pearl, 2009; Tenenbaum et al., 2011), neural-network based models have become the dominant approach in artificial intelligence, surpassing other approaches in a wide range of domains, including image recognition (Ciregan et al., 2012), language translation (Vaswani et al., 2017), and complex reasoning games like chess and go (Silver et al., 2016; Vinyals et al., 2019). Large language models (LLMs) (Bommasani et al., 2021) such as GPT-3 (Brown et al., 2020) are showing increasing proficiency in solving mathematical (Cobbe et al., 2021; Hendrycks et al., 2021; Lewkowycz et al., 2022; Uesato et al., 2022) and analogical problems (Webb et al., 2023). Some authors now claim that they are beginning to exhibit ‘artificial general intelligence’ (AGI) (Bubeck et al., 2023). However, LLMs have been found make consistency errors, such as failing to correctly answer ‘B is A’ when trained on ‘A is B’ (Berglund et al., 2023), and hallucinating invalid paths in planning tasks (Momennejad et al., 2023; Nam et al., 2022), raising questions on how well they understand and meaningfully use abstract rules.

It is likely that such models will continue to improve on benchmark assessments as newer versions of these models are released, but we believe that simply asking whether a machine can imitate or even surpass human-like responses on a battery of test questions—a variant of the Turing test (Turing, 1950)—is not an adequate measure for evaluating computational models of intelligence. Echoing the succession of behaviorism (Skinner, 1974; Watson, 1913) by the study of mental processes (Chomsky, 1957; Neisser, 1967) in the cognitive revolution, we see it as imperative to begin considering not only whether computational models can produce correct answers to large batteries of problems (Srivastava et al., 2022), but also the details of how they do so.

Thus, despite their achievements, we agree with Lake et al. (2017) that neural networks do differ from people in how they learn—there are key differences in the amount of data they require to learn and in their ability to generalize outside the range of examples seen in training. A comparison of the learning and generalization abilities of the successful solvers in our experiment with the performance of a recurrent relational network (RRN) model that achieves expert-level Sudoku capability (Palm et al., 2018) illustrates this important limitation (see SI Section 5). When trained on the restricted range of examples we used in the practice phase of our experiment, the RRN model requires on the order of one hundred thousand pattern presentations from a data set containing 300 unique problems, and fails completely when tested with target and distractor digits that had not been used in these roles during training. Similar limitations in a neural network based on the transformer architecture (Vaswani et al., 2017) can be observed when trained to solve Sudoku problems using a sequence of reasoning steps (Nam et al., 2022).

The approach to learning in these and similar network simulations is based on using a gradual, gradient-based learning procedure to adjust the connection weights in the network, a process that occurs slowly and generally requires interleaving of examples from the full distribution of the training data. Rapid learning from a few examples without interleaving can sometimes succeed if new examples align well with prior experience (McClelland, 2013), and some progress is now being made toward achieving out of distribution generalization with such methods (Abdool et al., 2023; Geiger et al., 2023, Lake & Baroni, 2023), but it is still true that these models require far more training data than the human participants who solved our hidden single puzzles within the first 3 to 10 trials of the practice phase of our experiment.

There is, however, now a different way in which neural networks can exhibit what has been called ‘few-shot learning’ (Brown et al., 2020). A model is said to exhibit few-shot learning if its performance on test questions is enhanced when it is given a sequence of relevant question-answer pairs in its context window, which plays a role similar to human working memory. However, this phenomenon may not always reflect new learning; in many cases it may simply prime the model to perform in accordance with the specific requirements of the question at hand, or to make superficial adaptations of its output to the task format (Min et al., 2022; Webson & Pavlick, 2022; Yadlowsky et al., 2023). Research addressing how and when networks rely on information in their context window is ongoing. It is now clear that transformer networks can learn to use information in context (Chan et al., 2022; Olsson et al., 2022), but this currently requires careful engineering of their training. Based in part on the findings from our experiments as well as work by others (Ahn et al., 1992; Chi et al., 1989; Lombrozo, 2006; Pearl, 2019; Rumelhart, 1980), we believe that one important part of helping models make better use of their context will be to find more human-like ways to encourage them to rely on explanations.

Previous efforts to train models to use explanations (Mishra et al., 2022), whether from scratch (Camburu et al., 2018, Lampinen, Roy, et al., 2022), through fine-tuning (Lampinen, Dasgupta, et al., 2022), or through conditioning with in-context prompts at evaluation time (Lu et al., 2022; Wei et al., 2022), have shown improved performance over models without explicit explanations. However, much of the existing literature remains largely empirical with limited theoretic accounts for the phenomenon (Xie et al., 2021). Successes to date with these approaches have been modest (Lampinen, Dasgupta, et al., 2022; Lampinen, Roy, et al., 2022), and further research will be required to enhance these capabilities.

Ultimately, given the evidence we reviewed above that advanced human cognitive abilities depend on mechanisms that allow us to organize our behavior in service of explicit goals, another important future direction will be to understand how to build models that are intrinsically goal directed (McClelland, 2022). As in the human case, this may involve both model architectures that enhance goal-directed behavior and better ways of helping models learn to be goal-directed through the experiences they receive during training.

## CONCLUSION

Our findings contribute both to understanding how humans learn and generalize abstract strategies for novel tasks and to identifying where humans and contemporary models diverge in these respects, offering insight on what behavior we ought to expect from a model that reasons as humans do beyond common measures of accuracy. Training models to hypothesize, explain, and to control their internal computations in service of meeting explicit goals would be important steps towards truly human-level reasoning, and finding ways to achieve these abilities will require fuller exploration in computational models. At the same time, many of the attributes of human reasoning that we have observed in our study remain incompletely understood. Continued effort to understand these human processes and to capture them in computational models offer exciting directions for future research.

## ACKNOWLEDEGMENTS

We thank Lucas Sato for being a rater for analyzing the participants’ self-reported strategies.

## FUNDING INFORMATION

Andrew Nam was funded by the NSF Graduate Research Fellowships Program.

## AUTHOR CONTRIBUTIONS

A.N.: Conceptualization; Data curation; Formal analysis; Investigation; Methodology; Project administration; Software; Validation; Visualization; Writing – original draft; Writing – Review & editing. J.M.: Conceptualization; Funding acquisition; Investigation; Methodology; Project administration; Resources; Supervision; Validation; Visualization; Writing – original draft; Writing – review & editing.

## DATA AVAILABILITY

The data for this paper can be found in the GitHub repository: https://github.com/andrewnam/systematic_human_learning.

## Note

^1^ This typo was made in the actual experiment.

## Supplementary Material


