# Supplementary material for: Systematic Human Learning and Generalization From a Brief Tutorial With Explanatory Feedback
Source: Open Mind (Camb). 2024 Mar 1;8:148–76. doi: 10.1162/opmi_a_00123 (PMC10898786; doi:10.1162/opmi_a_00123)

Supplementary Materials

Contents

1 Experiment design **47**
Diagnostic test and survey . . . 48
Puzzle generation . . . 49
Strategy survey . . . 50
Demographics survey . . . 52

2 Reported regressions **54**
Overall accuracy . . . 54
Accounting for tutorial house type . . . 55
Excluding random slopes . . . 55
Education . . . 55
Education model . . . 56
All math model . . . 56
Algebra and geometry model . . . 56
Education, algebra and geometry model . . . 56
All math and education model . . . 56

3 Unreported regressions **57**
Digit sets (DS) . . . 57
Goal position (GP) . . . 57
House index (HI) and cell index (CI) . . . 58
House type (HT) . . . 59

4 Questionnaire **60**
Multiple choice questions . . . 60
Free response ratings . . . 61

Participant selection 61
Rating options 61
Rating design 66
Rating consistency 70

5 Recurrent relational network 70

Model architecture 71
Replication of results 72
Solving hidden single puzzles 72
Inducing digit invariance 73

1 Experiment design

We describe important details about the experiment in this section, but the full experiment may be found in the GitHub repository (GITHUB_LINK_ANONYMIZED). Although the full code for the experiment is available, we also provide a PDF of screenshots in `figures/screenshots.pdf`. Note that the screenshots were generated for one possible participant and what might be shown at the start of each screen. The screenshots were taken within a development environment and contain artifacts that would not have appeared for actual participants, such as the accumulated compensation always being $0.00, the screen number, and "You solved out of 89 puzzles" on the final screen which should contain an actual number of puzzles solved. Some screens had short pop-up messages during the tutorial in response to participants' actions that are not depicted in the screenshots. Screens with the blue `Submit` button had a required task and most would not allow the participant to proceed unless they provided valid responses. Although the PDF shows screenshots for every screen in the experiment, not all screens were shown to all participants depending on their responses. We describe the control flow below.

1. Screen 2 (diagnostic survey): if the participant either correctly solved the diagnostic puzzle in Screen 1 and/or did not respond with "None" to the question "About how many Sudoku puzzles have you successfully completed?", skip to Screen 120.

2. Screen 5 (agreement): participants were allowed to proceed if their responses matched the prompt by at least 85%.

3. Screens 6-14 (tutorial): participants could only proceed after submitting correct responses (where relevant). Hints were provided through short pop-up messages if they submitted incorrect responses.

4. Screens 18-42 (practice phase): participants could only proceed after inputting the correct digit in the goal cells.

5. Screens 44-107 (test phase): if participants correctly solved a puzzle, they were allowed up to 10 seconds before automatically proceeding to the next screen (this period may be skipped). If they submitted an incorrect response (including a blank) or did not submit anything for 2 minutes, they were required to wait 10 seconds before automatically proceeding to the next screen.

6. Screen 108 (questionnaire attention check): participants could only proceed after answering all 3 questions, regardless of correctness.

7. Screens 109-119 (questionnaire): see Section 4

The experiment program was implemented using Facebook's React, hosted on Amazon AWS, and deployed using Psiturk (Eargle et al., 2021; Gureckis et al., 2016).

Diagnostic test and survey

At the beginning of the experiment, in order to filter out any participants who had prior experience in solving Sudoku puzzles, we presented a simple diagnostic test and a survey. Participants were not informed about the purpose of the diagnostic material.

The diagnostic puzzle was a 4x4 Sudoku grid as shown in Figure 11. Participants were told that the puzzle was a 4x4 variant of Sudoku and were instructed in how to interact with the program interface. They were offered $0.25 to complete the puzzle with a $0.01 penalty for each incorrect attempt. All cells needed to be correct for the puzzle to be considered solved.

After the diagnostic puzzle, participants were presented with the following survey:

1. Have you heard of Sudoku before?
   (a) Yes
   (b) No
   (c) Not sure

2. Have you ever attempted to solve a Sudoku puzzle?

    (a) Yes

    (b) No

    (c) Not sure

3. About how many Sudoku puzzles have you successfully completed?

    (a) None

    (b) 1 to 3

    (c) 4 to 6

    (d) 7 to 9

    (e) 10 or more

Only participants that did not solve the diagnostic puzzle and also responded that they had never successfully completed any Sudoku puzzles were proceeded to the rest of the experiment. For all others, the program skipped to the demographics survey and terminated thereafter. Of the 1,985 people that originally entered the study, 1,384 successfully solved the diagnostic puzzle and 1,668 responded that they had completed one Sudoku puzzle in the past, leaving 271 participants in neither group to complete the experiment.

Puzzle generation

All puzzles used in the experiment were generated with the following procedure according to the specified house type, goal cell, and digit set.

First, from the digit set of four numbers, we selected one as the target and one as the distractor. From the remaining seven, we selected three as the in-house digits. We chose one of the 2 boxes that intersect with the target house but not containing the goal cell to contain one target digit and one distractor digit. The three cells in this box intersecting the target house were left empty. Of the remaining 5 non-goal cells in the target house, we selected

three to contain the three in-house digits. Of the remaining 2 cells, we selected one to contain both the target and distractor cell in its row or column (whichever is orthogonal to the target house), with the other containing only the target digit. The third distractor was placed anywhere in the grid such that it would intersect with the target house only in cells that already could not contain the distractor. All selections were made randomly subject to the Sudoku constraints.

One instance is placed to constrain three empty cells in the target house by sharing the same box as them, and another to constrain a single empty cell by sharing a column or row (whichever is orthogonal to the target house) with it. The third target digit instance forms a second orthogonal constraint, which is what forces the goal cell to be the only remaining cell in the target house that could contain the target, whereas the distractor digit is placed to allow two cells in the target house that could contain it. 3 cells in the highlighted house were filled with random remaining digits. Because this would require at least 3 hints that share digits with the target, we added a distractor digit with 3 hints that constrain the same box and one of the 2 other unconstrained cells, making both the target and distractor digits salient as potential candidate target digits.

Strategy survey

After completing the text phase described in the main text, participants were first instructed on the contents of the following segment and asked 3 attention-check questions. Next, they were given a puzzle sharing the same house type and digit set as the tutorial, but with the goal cell located in the center box and were asked to select and enter the digit that must go in the goal cell. Without providing feedback on correctness, we then displayed the puzzle and their response for the remainder of the questionnaire, allowing the participants to refer to it as necessary. We asked the following questions in order of increasing specificity using free-response questions to elucidate general responses without additional prompting and more specific multiple-choice questions to formalize their strategies.

1. "How confident do you feel that your answer is correct, expressed as a percentage?"

(Responses were allowed between 0 and 100 in increments of 5.)

2. "Explain as clearly as possible the steps you went through to choose your answer. Please be as detailed as possible so that someone else could replicate your strategy by following your response."

3. "There are two numbers in the puzzle that occur three times outside of the row/column containing the target cell. Which of the following best describes how you chose between the two candidate numbers to consider?"

   (a) I noticed something in the puzzle that initially made one candidate seem more likely to be correct than the other.

   (b) I arbitrarily chose between the two candidates because they seemed equally promising to consider.

4. "What did you notice in the puzzle that initially made one candidate seem more likely to be correct than the other?"

5. "Please select the cell(s) that initially made one candidate seem more likely be correct than the other." (Participants could select one or more cells in the grid.)

6. "Please explain how the cell(s) you selected initially made one seem more likely to be correct than the other." (Previously selected cells were shown.)

7. "After you selected a candidate to consider, did you check further to determine whether that candidate was actually correct or not?"

   (a) Yes, I checked to see whether the candidate was actually correct.

   (b) No, I just submitted my original guess without checking any further.

8. "What did you do to determine if that candidate was actually correct?"

9. "Which of the following best describes the way you determined whether or not the candidate was actually the correct answer?"
   (a) I checked whether the candidate I chose could go in any of the empty blue cells in the row/column.
   (b) I looked at other numbers in the puzzle until I noticed something that helped me decide whether or not the candidate was correct.
10. "Please provide any additional information or clarifications to any of your previous responses so that we can most accurately understand as best we can how you solved this puzzle."

Participants that did not respond to the puzzle with the target or dstractor skipped ahead to Question 10 after Question 2. Participants that responded with option (b) in Question 3 skipped ahead to Question 10. Participants that responded with option (b) in Question 7 skipped ahead to Question 10.

Demographics survey

Following the strategy questionnaire, we also asked the participants about their demographic information including their age, gender, highest level of education, and mathematical topics they have taken courses in. All participants, regardless of diagnostic test and survey results, were asked the following questions about their educational backgrounds. Participants that were filtered out from the diagnostics were presented the demographics survey immediately after the diagnostic survey screen.

"What is your highest level of education (including currently pursuing)?" Participants were allowed to select one of the following:

- Have not graduated high school
- High school graduate, diploma or equivalent
- Associate degree

- Bachelor's degree
- Master's degree
- Professional degree (e.g. M.D., J.D.)
- Doctoral degree

"Degree status" Participants were allowed to select one of the following:

- Currently pursuing
- Completed

"Which of the following mathematics topics have you taken a course in? Select all that apply." Participants were allowed to select zero or more of the following:

- High school algebra
- High school geometry
- Trigonometric functions
- Single-variable calculus
- Multi-variable calculus
- Linear algebra
- Probability & statistics
- Discrete mathematics
- Formal logic

2 Reported regressions

Here, we provide the exact formulas used in the regressions reported in the main text and the full list of fitted coefficient values. All regressions were fitted using the BRMS package in R (Bürkner, 2017) with `adapt_delta` = .99, `iter` = 10000, and otherwise default priors and MCMC settings. For any regressions that had any divergent transitions, we increased `adapt_delta` to .999, at which point no divergent transitions were reported. Otherwise, we followed the diagnostic recommendations as provided in the 'Runtime warnings and convergence problems' page by the Stan development team https://mc-stan.org/misc/warnings.html, such as verifying that `Rhat` $\leq$ 1.01 and `Bulk_ESS` $\geq$ 400 (using 4 chains). All reported coefficients on accuracy models are in logits. All reported coefficients on response time models are in $\log_2$(seconds). We report 95% highest density credible intervals for the parameter estimates.

Only trials with correct responses were used to fit the response time models. In each model, we accounted for improvements through practice using a $\log_2$(trial) term and for individual variations through random effect intercepts for each participant. t refers to the trial number (between 1 and 25 for the practice phase, between 1 and 64 for the test phase) and s refers to the subject. The $(1 + \log_2 t|s)$ included in each regression indicates participant-level random effects. All other terms are fixed effects.

Overall accuracy

We used Bayesian logistic mixed-effects models for predicting the correctness of each trial for both phases. We note that the preregistered model was not Bayesian, but we chose to change the method for consistency with the remainder of the paper. These models were only used for classifying solvers and non-solvers, and all classifications were consistent between both modeling approaches.

The null models we used for comparison in analyzing the test phase results are

$$P(\text{correct}_{t,s}) \sim \log_2 t + (1 + \log_2 t|s)$$

$$\log(\text{duration}_{t,s}) \sim \log_2 t + (1 + \log_2 t|s)$$

Tables 2, 3, 4, 5, 6, and 7 show the fitted coefficients to the test phase regressions. Figures 12 and 13 show the posterior samples of coefficients from the fitted models.

Accounting for tutorial house type

We checked to see if the effects were moderated by whether the participants were taught the hidden single technique using rows or column. Specifically, we added terms C indicating whether the participant's tutorial used columns and $HT \times C$.

$$P(\text{correct}_{t,s}) \sim HT + C + HT \times C + \log_2 t + (1 + \log_2 t|s)$$

$$\log(\text{duration}_{t,s}) \sim HT + C + HT \times C + \log_2 t + (1 + \log_2 t|s)$$

None of the models fit the data significantly better than the house type model without C and $HT \times C$. The Bayes factor for the accuracy model was 1.43 for the first 16 trials and 0.60 for the last 48 trials. The Bayes factor for the RT model was 0.06 for the first 16 trials and 0.10 for the last 48 trials. The fitted coefficients are in Tables 8 and 9.

Excluding random slopes

As noted in the main manuscript, we did not see meaningful differences in model estimates and credible intervals when excluding random slopes. Table 10 shows the same regression coefficients and CIs as Table 1 comparing models with and without random slopes.

Education

In the questionnaire, we asked for highest education pursued, whether completed or in-progress. However, because we found that some education levels were extremely rare in our dataset (e.g. PhD), we converted them into years of education with the following mapping: *Incomplete High School* → 10, *High School* → 12, *Associate's Degree* → 14, *Bachelor's Degree* → 16, *Master's Degree* → 18, *Professional Degree* → 20, *PhD* → 21.

Education model

$$\text{num\_solved} \sim \text{education}$$

All math model

$$\text{num\_solved} \sim \text{alg} + \text{geom} + \text{trig} + \text{sv\_calc} + \text{mv\_calc} + \text{linalg} + \text{pr\_stat} + \text{disc} + \text{logic}$$

Algebra and geometry model

$$\text{num\_solved} \sim \text{alg} + \text{geom}$$

Education, algebra and geometry model

$$\text{num\_solved} \sim \text{education} + \text{alg} + \text{geom}$$

All math and education model

$$\text{num\_solved} \sim \text{education}+\text{alg}+\text{geom}+\text{trig}+\text{sv\_calc}+\text{mv\_calc}+\text{linalg}+\text{pr\_stat}+\text{disc}+\text{logic}$$

3 Unreported regressions

Here, we describe the regressions that were not included in the main article but were committed in the preregistration. Due to the large number of parameters, we do not include their estimates here. For the coefficients of these models, see the spreadsheet in the project repository.

As in the previous section, t refers to the trial number (between 1 and 64) and s refers to the subject. The $(1 + \log_2 t|s)$ included in each regression indicates participant-level random effects. All other terms are fixed effects.

Digit sets (DS)

One additional regression was conducted for the digit sets analysis which included an interaction term between the treatment and practice effects. This model was fitted using data from all 64 test phase trials.

$$P(\text{correct}_{t,s}) \sim DS + log_2 t + DS \times log_2 t + (1 + log_2 t|s)$$

$$\log(\text{duration}_{t,s}) \sim DS + log_2 t + DS \times log_2 t + (1 + log_2 t|s)$$

Goal position (GP)

One additional regression was conducted for the goal position analysis which included an interaction term between the treatment and practice effects. While this model was not committed in the preregistration, we include it for completeness. This model was fitted using data from all 64 test phase trials.

$$P(\text{correct}_{t,s}) \sim GP + log_2 t + GP \times log_2 t + (1 + log_2 t|s)$$

$$\log(\text{duration}_{t,s}) \sim GP + log_2 t + GP \times log_2 t + (1 + log_2 t|s)$$

House index (HI) and cell index (CI)

These regressions were the original formulations for analyzing the effect of moving the goal cell. Due to the transposition of the grid when applying the house type (HT) condition and the high perceptual dissimilarity, we intended to perform separate regressions for puzzles with the HT condition applied and for puzzles without the HT condition applied. However, post preregistration, we had decided that the effect of interest was less about exactly which axis the goal cell had translated across and more about the presence of a change at all. Moreover, we had originally planned to account for an interaction between HI and CI, but decided that the interaction complicated the interpretation. Thus, we collapsed house index and cell index conditions as the goal position (GP) condition in favor of improved power and interpretability, considering the condition to be applied if one or both of the house index or cell index had been applied.

Note that each of the four regressions below have been fitted separately using trials with and without house type conditions.

16-trial and 48-trial models

$$P(\text{correct}_{t,s}) \sim HI + CI + HI \times CI + (1 + log_2 t|s)$$

$$P(\text{duration}_{t,s}) \sim HI + CI + HI \times CI + (1 + log_2 t|s)$$

64-trial models

$$P(\text{correct}_{t,s}) \sim HI + CI + HI \times CI + HI \times log_2 t + CI \times log_2 t + HI \times CI \times log_2 t + (1 + log_2 t|s)$$

$$P(\text{duration}_{t,s}) \sim HI + CI + HI \times CI + HI \times log_2 t + CI \times log_2 t + HI \times CI \times log_2 t + (1 + log_2 t|s)$$

House type (HT)

One additional regression was conducted for the house type analysis which included an interaction term between the treatment and practice effects. This model was fitted using data from all 64 test phase trials.

$$P(\text{correct}_{t,s}) \sim HT + log_2 t + HT \times log_2 t + (1 + log_2 t|s)$$

$$\log(\text{duration}_{t,s}) \sim HT + log_2 t + HT \times log_2 t + (1 + log_2 t|s)$$

4 Questionnaire

Multiple choice questions

Comparing the responses between the persistent-solvers and PD-guessers, we found significant differences across all 3 questions. Specifically, when asked about how they chose which of the two prevalent digits they considered, 80.95% of persistent-solvers and 58.33% of PD-guessers responded that they had noticed something in the puzzle that made one candidate seem more likely versus chose arbitrarily. Among those that noticed something, 75.00% of persistent-solvers and 45.90% of PD-guessers responded that they had further checked to see if their chosen candidate was correct versus submitted without checking. Finally, among those that checked, 72.55% of persistent-solvers and 36.36% of PD-guessers responded that they checked to see if the candidate could go in another cell in the house versus looking for information in other numbers. Figure 14 and Table 16 show group means and 95% CI for each question.

Free response ratings

Participant selection

For our ratings of questionnaire results, we focused our analysis on subsets of the solver and non-solver groups based on test phase performance. Among the solvers, we selected a subset we call *persistent solvers* that demonstrated high accuracy at the end of the test phase according to a logistic regression model fitted to the test phase data, similar to the logistic regression fitted to the practice phase data in the original solver classification. 84 solvers that had a predicted accuracy of at least 80% on the 64th trial of the test phase were classified as persistent solvers and were included in the strategy ratings.

Among the non-solvers, we focused on a subset we call *PD-guessers* that exhibited evidence of consistently guessing between the target and distractor. PD-guessers were defined as non-solvers that had a predicted accuracy of at most 60% at the end of the test phase according to the logistic regression model, had solved 3 to 5 of the last 8 puzzles in the test phase, and had selected the target or the distractor in at least 58 of the 64 test phase puzzles. 84 of 183 non-solvers were identified as PD-guessers and were included in the strategy ratings.

Rating options

Here, we show the options available to the raters, as well as a selected response from participants for each category. All examples shown were rated by both raters to belong in the specified category.

Awareness of error: The following options were available for identifying whether or not the responses indicated awareness of error. This was only done for puzzles that were not solved correctly.

1. **Yes with explanation:** The participant indicates a clear realization that the answer they entered was not correct, and explains why they decided that the other answer was correct.

"I actually realize now that I made a mistake while solving this puzzle. I think I should have put a 2 where I put the 9. However my strategy was to check each blue square, seeing if either of the numbers in the white boxes would fit there. In each case there was one of the numbers that could only go one place while still leaving a spot for the other number."

2. **No or yes w/o explanation:** The participant's response was incorrect, but they did not explicitly report realizing that they had answered incorrectly, or the response suggests they think they were wrong without certainty or clarity about the reason.
"Whatever third of the puzzle contained two non-blue numbers, I used those two numbers as a choice. Whichever would fit in a blue square exclusively was right."

Prevalent digits: The following options were available for identifying prevalent digits mentioned in the responses.

1. **Both:** The participant mentioned both prevalent digits by name, or otherwise led you to believe they realize that they have to choose among these two digits.
"I went through a process of elimination by checking which numbers were already included in each square, since you can't duplicate numbers. But to be honest, I'm not sure if the 1 should go in the 1st, 3rd, or 4th blue box, Or if the 4 is in the 2nd, 3rd, or 4th. Sadly, these puzzles have made me feel not very bright!"

2. **Target:** The participant mentioned the target, by name or in some other way, but did not mention the distractor, and did not indicate that there were two prevalent digits.
"saw which number couldn't be elsewhere in the highlighted column"

3. **Distractor:** The participant mentioned the distractor, by name or in some other way, but did not mention the target.
"Well I just chose what i felt was the best possible answer and I thought 4 was the one that fit the most in that square. Sometimes you just have to go with your gut."

4. **Neither:** The participant did not mention either the target or the distractor, either by name or some other way, and there is no indication of awareness that there are two prevalent digits.
   "I just calculating the values in the first row and accordingly I have selected this"

5. **Vague or uncertain:** The participants' answer could be signaling that they were selecting between the two prevalent digits or that they were focusing on a digit that cannot go elsewhere or the distractor, but they do so vaguely or in a way that it is hard to be certain.
   "Since I'm still not very knowledgeable about Sudoku, I just looked at which number was farthest away from the empty box and chose it."

Basis of choice: The following options were available for identifying the basis for choosing between the two prevalent digits. Figure 15 shows the distributions of ratings for solvers and non-solvers.

V1 Chose digit that cannot go elsewhere: The participant states that a digit cannot go in any of the empty blue cells and gives that digit as the answer. As a shortcut variant of category A, participant states that they found a cell in the target house that was only constrained by one of the two prevalent digits and chose that digit as the answer. To be valid, this must be a cell that is not in fact constrained by the distractor.
"I located the larger square with two numbers in it (6 and 1) that also contained three small blue squares. This told me the green square would have a 6 or 1. I then checked to see if 6 could fit in one of the two remaining blue squares. Since there was already a 6 in each row I knew 6 had to be the solution for the green square."

V2 One PD can go elsewhere, chose the other: The participant states that one of the prevalent digits can go in one of the empty blue cells and concludes that the other prevalent digit is the answer.

"First, I eliminated the quadrant with both the 3 and the 1 as a means of determining which number to pick. Then I looked at the quadrant with the two empty boxes and mentally interrogated the empty boxes. Since I new that one empty box could not contain a 3 or a 1 as these were both in the row containing the box, I moved on to the other empty box. I reasoned that since the last box had a row containing the 3, I could not eliminate 1 as potentially populating this box. The empty green box then, should contain a 3."

V3 Found cell where one PD could go and the other could not: The participant mentions finding a blue cell that can contain one of the two prevalent digits and not the other, and chooses the digit that cannot go in that cell as the answer.

"So i find the square that had the 7 and 5 already in it I know since that has a 7 and 5 that I can eliminate those 3 blue squares. Then I go to the green square and find which blue square that can't be a 7 or 5 and then i find the blue square that can't be a 5 but can be a 7 so then I know since that blue square would be a 7 our green square would be the 5"

U1 Potentially valid but general or not fully specified: The participant's response provides incomplete information about how they came to choose their answer, but could be a general or under-specified description of a valid procedure.

"I look at the possible solution numbers. Next I look at each empty cell and ask myself if a possible solution number fits into the empty cell as the correct answer I am looking for."

U2 Unclear, confused or missing basis: The answer attempts to provide information about how the choice was made, but is unclear, incorrect, confusing, or fails to specify the procedure used to select between the PDs.

"Whatever third of the puzzle contained two non-blue numbers, I used those two numbers as a choice. Whichever would fit in a blue square exclusively was right."

I1 Explicit guess: The participant indicates that they know they are guessing (either completely at random or between the two prevalent digits)
"I mostly guessed. I'm still learning how to play this game."

I2 Irrelevant basis for choice: The participant indicated a basis for choosing the answer that was not related to the logic of the puzzle.
"my strategy is very simple , add these numbers by the given number outside of the box if it is 15 i choose lowest number if its 19 or above i choose higher number , mostly i choose randomly"

I3 Chose the most frequent digit: The participant indicates they chose the digit that occurred the most frequently in the puzzle, apparently not realizing that there were always two digits that both occurred exactly 3 times.
"I looked at the number (7) that is duplicated in most of the cells and usually picked that one."

M Did not answer the question: Response does not address the question.
"yes"

O Other: None of the above.

The shortcut variant of option V1 was one heuristic that we observed that did not clearly fit into one of the four strategies outlined in the results section. As an illustration, take one solver's response after having correctly solved the left puzzle in Figure 16: "The first thing I did was locate the square section that contains both of the possible numbers and the three empty blue squares. After locating this section, I can completely ignore this area since it is irrelevant to finding the answer now. Next I look for either of the two possible numbers (in this case 2 or 3) which is isolated, in a row or column all by itself. Upon finding that number, I'm finished. Just take that number, and since you know that its counterpart will be filled in in the blue row, you can just take the isolated number and make it your answer."

This description is a valid method of arriving at the target digit for the given puzzle, but is not a valid strategy as it is not guaranteed to work in all the hidden single puzzles, such as the right puzzle in Figure 16. In this case, the 3 on the bottom row and the 4 on the top row are both sole occupants of their respective columns so that both the distractor and the target digits meet the "isolated number" criterion. This strategy could be made valid by checking that the cell in the blue highlighted house that the 3 intersects with is also constrained by a 3 in its 3x3 box and selecting the other prevalent digit as the target. The method used to generate the hidden single puzzles has a 40% chance to produce puzzles with these redundantly constraining distractors. If one exclusively uses this strategy and randomly guesses between the 2 prevalent digits when this situation occurs, the expected accuracy would be 80%, which coincidentally is also the value of the decision boundary we used to classify solvers and non-solvers at the end of the practice phase. Of the 84 persistent-solvers' responses examined, only 6 were noted to be describing a strategy that resembled this heuristic.

Rating design

One author (ANON2) went through all 168 persistent solvers' and PD-guessers' responses to the first free-response question in an effort to develop a set of categories into which individual participants' responses could be sorted, with access to the specific puzzle each participant had just attempted to solve, the participant's chosen response, and summary scores characterizing the participant's performance both on the practice and test phases of the experiment. In so doing, he noted that persistent solvers typically (a) referenced the target digit or both the target and the distractor; (b) provided one of three types of responses that seemed to describe a valid solution strategy given the rules of Sudoku and further constraints on the constructions of the puzzles used, and, (c) in the small number of cases in which a solver incorrectly chose the distractor, explicitly reported realizing that they had selected the wrong digit and explained why their choice was wrong. The author also noted that non-solvers were (a) less likely to mention either or both

prevalent digits even if they almost always chose one of the two prevalent digits, (b) rarely described a valid solution strategy, and (c) in the cases where they chose the distractor, never expressed a clear understanding that the response was incorrect. The author began to develop a set of scoring criteria that could be used to corroborate these impressions. He developed preliminary versions of the awareness of error, mention of prevalent digits, and basis for choice rating categories, including preliminary versions of the set of options for raters to choose among with made up illustrative examples. The other author (ANON1) checked the categories and descriptions for clarity and distinctness with minimum reference to the actual data. The two authors then met to refine the categories, their short names, their longer descriptions, and the illustrative examples in an attempt to span the range of variation of responses that fell into each of the categories. The authors jointly agreed on the criteria used to identify the persistent solvers as a subset of the solvers and the PD-guessers as a subset of the non-solvers. Note that this excluded several individuals who might have been late or partial solvers as well as others who chose responses other than one of the two PDs on more than 6 (corresponding to more than 10%) of the test trials.

Next, both authors rated a pilot set of 20 participants' responses. As with the final ratings discussed below, these ratings were carried out with access to the specific puzzle each of the participants solved and the answer the participant gave, but without reference to information about the participant's classification as a persistent solver or a PD-guesser or any other information about the participant including their performance in the training or test phases of the experiment. To make the pilot set representative without inspecting the actual responses, we sampled 9 *persistent solvers* that solved the puzzle, 1 *persistent solver* that did not solve the puzzle, 6 *PD-guessers* that solved the puzzle, and 4 *PD-guessers* that did not solve the puzzle. After independently rating all 20 participants, the authors further discussed and refined the categories, descriptions, and examples. In this process we noted that some responses were difficult to categorize with certainty, but that in these cases the uncertainty was restricted to two alternative possibilities. Accordingly we adjusted the

scoring system to allow raters to provide a second choice category to capture these cases.

For the final ratings, we wished to measure the extent to which persistent solvers (a) referenced the target digit or both the target and the distractor in their response to the first free response question; (b) indicated that they had employed one of the three valid solution strategies; and (c), reported a realization of their error and explanation of why it was wrong on cases where they entered the distractor as their response instead of the target. We wanted to demonstrate that this determination could be made reliably and in the absence of any other information beyond the specific puzzle the participant received during the questionnaire phase, the digit choice the participant entered as their solution, and their response to the first free response question about how they solved the problem. We further wished to measure the extent to which PD-guessers exhibited these same tendencies, and if not, to document the distribution of responses they provided related to all three of these questions. Because author ANON2 had previously considered all of the participants responses with additional information, this author did not contribute to the final ratings. Instead we sought an individual who would be motivated to work conscientiously to identify the strategy used by each participant without having had any opportunity to consider any other information about each participant beyond the specific puzzle the participant saw, their digit choice, and their answer to the first free response question. A Stanford computer science Master's student (ANON3) pursuing research on human problem solving in collaboration with author ANON2 met this criterion. ANON3 had participated in lab meetings in which the behavioral choice results from the study had been discussed, including the classification of participants as solvers or non-solvers, but had not been exposed to details of the performance of any of the participants. ANON3 and author ANON1 then served as the two raters for the full set of participants. Although ANON1 had previously considered some of the participants free responses with other information about their performance, we reasoned that if agreement between the two raters was high, this would be a sufficient indication that the ratings could be made reliably without access to additional

information about the participants. ANON3 was paid $30 per hour for his participation. He first completed the experiment to familiarize himself with the study, and was classified as a persistent solver, achieving 100% accuracy in the practice phase and 100% accuracy in the test phase of the experiment. He then reviewed the rating instructions and guide thoroughly before rating the 20 pilot participants. Subsequently ANON2, ANON1, and ANON3 met to discuss the ratings criteria and discrepancies between ANON1's and ANON3's ratings of these participants. As a result of this discussion we made some final refinements in the category descriptions and examples until all three were satisfied that they were as clear as possible and that all three had a common understanding of the categories. ANON1 and ANON3 then adjusted their ratings in accordance with the refined categories. We did not require the two raters to necessarily agree perfectly on their first choice rating category, allowing disagreements to reflect the ambiguities that were present in some of the responses.

Finally ANON1 and ANON3 proceeded to rate the responses of the remaining 148 persistent solvers and PD-guessers. To rate each participant, the rater first looked at the specific puzzle the participant had received and judged whether or not the participant's response was correct (all of these participants choices were one of the PDs). The rater entered this judgment in a spreadsheet, which then checked whether the rater's judgment of the participant's response was correct and alerted the rater in the rare case that the rater was incorrect in judging the correctness of the participant's response (this occurred 0 times for rater ANON3 and 1 time for ANON1). The rater proceeded to rate the participants' free responses, proceeding through the ratings in the order listed above. Raters were asked to complete their ratings in several sessions spread out over a one-week period, and to proceed slowly and to work for no more than an hour at a single sitting, though they could resume work after a short break. Raters were also asked to check their ratings and to reconsider cases where they were initially uncertain, and were allowed to adjust any of their ratings after completing a first pass rating the full set. We expected there would be some cases of inconsistency in the ratings, and did not seek to resolve these inconsistencies, so that for all

but 20 of the rated participants, the final ratings of the two raters were reached without influence from the other rater.

Rating consistency

For each participant, as an attention check, raters first determined whether or not the participant's answer to the puzzle was correct. Both raters scored very highly on this with accuracies of 97% and 100%. Raters were also asked to determine whether or not the incorrect participants expressed awareness of their errors. Of the 4 persistent solvers and 42 PD-guessers that did not correctly solve the puzzles, both raters agreed on all 46 identifications. The two raters had 89.3% agreement on which PDs were mentioned by the participants (Figure 17).

Identifying bases for choice was overall a much tougher task, often due to incomplete ideas or ambiguous phrasing from the participants. Even when the responses were clear and relatively easy to understand, the different valid bases could be viewed as different ways of approaching the same logic, making classification difficult for some responses. Moreover, some participants mentioned multiple strategies, sometimes accounting for counterfactual scenarios where the first PD they considered ended up being the distractor but acknowledging it could just as likely have been the target. Therefore, we allowed a second option in case a response mentioned multiple legitimate strategies or was borderline unclear.

At the superclass level, the raters had 91.7% agreement for the solvers and 82.1% for the non-solvers, with an overall agreement of 86.9%. At the subclass level, the raters had 64.3% agreement for the solvers and 75.0% for the non-solvers, with an overall agreement of 69.6%.

5 Recurrent relational network

To compare how contemporary neural network models learn and generalize solving the hidden single puzzles, we replicated and adapted a model that is state-of-the-art in solving Sudoku puzzles (Palm et al., 2018). The recurrent relational network (RRN) uses a relational message passing scheme where in each time step, it computes for each cell in the

grid an update instruction for each other cell that the cell shares a house with. The model achieves systematicity with respect to the positional variables by sharing the same connection weights to each cell from all of the relevant constraining cells, thereby remaining invariant to the particular cell it solves for and the particular relevant cell constraining it.

Model architecture

The recurrent relational network uses a local message passing scheme in a graph where each cell in the Sudoku grid is a separate node. Cells that share houses (row, column, or box) are considered neighbors and their nodes share edges between them. Each node i at time step t is represented by a hidden state vector h_i^t, where $h_i^1 = x_i$ is an embedding of the cell's initial state (e.g. blank or a clue). In the original paper, the initial cell state is given by

$$x_i = \text{MLP}(\text{concat}(\text{embed}(d_i), \text{embed}(\text{row}_i), \text{embed}(\text{column}_i))) \tag{12}$$

where d_i is the initial content of the cell, if any. In our implementation, we found the cell coordinate information detrimental to performance and simplified the embedding to $x_i = \text{embed}(d_i)$.

At each step, for each cell i and its neighbor j, a vector representing the message from j to i is computed using

$$m_{ij}^t = \text{MLP}(\text{concat}(h_i^{t-1}, h_j^{t-1})) \tag{13}$$

Then the messages from all of i's neighbors are summed as

$$m_i^t = \sum_j m_{ij}^t \tag{14}$$

The subsequent hidden state vector of each cell is updated using

$$h_i^t, c_i^t = \text{LSTM}(\text{MLP}(\text{concat}(x_i, m_i^t)), c_i^{t-1}) \tag{15}$$

In our implementation, however, we found a simple linear layer to be sufficient replacements for the two MLPs.

An output vector is also calculated for each step and cell using a linear decoding layer

$$o_i^t = \text{linear}(h_i^t) \tag{16}$$

which is then used to calculate the cross-entropy loss with the solution for the cell.

Replication of results

We attempted to replicate the findings of Palm et al. using the same dataset and parameters as the original network. However, due to limited computational resources, we had to use our simplified architecture and fewer parameters. Specifically, we used a digit embedding size of 10 (9 digits + blank; original model used 16) and reduced the MLPs to single linear layers. Moreover, we reduced the number of training epochs to 100 and the batch size to 20. Our replication attempt resulted in 97.0% of all cells solved and 74.2% of test puzzles fully solved for all 81 cells. While this is much lower than the 96.6% reported in the original paper, it demonstrates that our simplified architecture can achieve a significant level of success while retaining key features of the original architecture.

Solving hidden single puzzles

To compare the model to human performance, we trained and tested the RRN model using the hidden single puzzles similar to what we provided for our participants. Specifically, we created a set of puzzles with the same digit set, house index, cell index, and house type to train the model. We then generated 64 puzzles with varied features to test for the model's ability to generalize. Because the majority of the grid was empty, we only calculated the cross-entropy loss for the goal cell and the initial hint cells. We found the latter to be a critical auxiliary signal for the model to successfully train.

The model was written using PyTorch and trained using Adam with batch size = 100, learning rate = 0.001 (original model used 0.0002), and L2 regularization of 0.0001. We used

a digit embedding size of 10 and a hidden layer size of 48 (original model used 96). We found these parameters sufficient given the significantly lower complexity of the hidden single puzzles compared to full Sudoku puzzles.

Using the same set of restrictions on the practice puzzles as described in our human experiments, we trained the model with varying numbers of training samples, stopping if and when it reached 99% accuracy for puzzles with the same features. We then tested the trained model with the same systematic variations we considered for human participants. Comparing the model's training and generalization results to human solvers, we observed two key properties of the network.

First, as shown in Figure 22a, the model trained inefficiently compared to the human participants, requiring 300 unique puzzles and on the order of a hundred thousand of total pattern presentations to gradually reach accuracies comparable to the solvers. Second, given the same restrictions on the particulars of the training puzzles as described in our human experiments, the model performing nearly perfectly on problems with changes to the positional features (house type, house index, and cell index) but could not solve a single puzzle with changed digit sets (Figure 22c).

Inducing digit invariance

If weight sharing across spatial features produces spatial invariance, it stands to reason that weight sharing across the digit feature can produce digit invariance. Following this logic, we expanded the Palm RRN by defining a node for each cell and digit (x, y, digit), thus producing $9^3 = 729$ nodes. We defined edges between any two nodes that represent different digits of the same cell or two nodes that represent the same digit of two cells sharing a house. For example, nodes for (3, 3, 1) and (3, 3, 6) would share an edge because they both represent the cell at (3, 3). Nodes for (3, 3, 9) and (3, 6, 9) would share an edge as they share the same house at Row 3 and the same digit, but (3, 3, 7) and (3, 6, 9) would not share an edge since they do not share the same digit.

Since individual nodes represent digits, we no longer needed a layer to embed

numbers. The output layer was also modified such that each node maps directly to a scalar logit, and the 9 digit nodes of a cell were then passed to the softmax function to convert these logits into digit probabilities. Due to memory resource constraints, the hidden state and message vector sizes were reduced to 16 and the batch size reduced to 10. We refer to this variant of the RRN as the digit-invariant recurrent relational network (DRRN).

Adding this inductive bias strongly constrained the solution space of the model such that it could reach near perfect accuracy using as few as 25 training examples, but still required thirty to seventy thousand training examples to achieve a high level of accuracy (Figure 22a). In addition, the DRRN was often unstable during training, possibly due to its low-dimensional hidden vectors. However, when we evaluated performance on held-out test puzzles on epochs where the models had the highest validation accuracy, we found that the DRRN successfully generalized as well to held out training digits as it did in the other three feature variations (Figure 22c).

Table 2

Digit set: accuracy regression parameter estimates. Reported numbers in logits.

	Trials 1-16			Trials 17-64		
Term	Estimate	CI-L	CI-U	Estimate	CI-L	CI-U
intercept	2.41	1.74	3.23	1.82	0.27	3.38
$\log_2$(trial)	0.09	-0.15	0.31	0.28	-0.01	0.58
DS	-0.10	-0.46	0.26	0.02	-0.24	0.28

Table 3

Digit set: duration regression parameter estimates. Reported numbers in log_2 seconds.

	Trials 1-16			Trials 17-64		
Term	Estimate	CI-L	CI-U	Estimate	CI-L	CI-U
intercept	4.45	4.30	4.61	4.60	4.28	4.92
$\log_2$(trial)	-0.13	-0.17	-0.09	-0.19	-0.24	-0.13
DS	-0.02	-0.09	0.04	0.00	-0.03	0.03

Table 4

Goal position: accuracy regression parameter estimates. Reported numbers in logits.

	Trials 1-16			Trials 17-64		
Term	Estimate	CI-L	CI-U	Estimate	CI-L	CI-U
intercept	2.23	1.53	3.04	2.06	0.52	3.60
$\log_2$(trial)	0.28	0.00	0.58	0.092	-0.030	0.209
GP	0.31	-0.65	0.01	-0.314	-0.668	0.019

Table 5

Goal position: duration regression parameter estimates. Reported numbers in log_2 seconds.

	Trials 1-16			Trials 17-64		
Term	Estimate	CI-L	CI-U	Estimate	CI-L	CI-U
intercept	4.35	4.19	4.52	4.53	4.22	4.85
$\log_2$(trial)	-0.13	-0.17	-0.09	-0.19	-0.24	-0.13
GP	0.11	0.04	0.19	0.09	0.05	0.13

Table 6

House type: accuracy regression parameter estimates. Reported numbers in logits.

	Trials 1-16			Trials 17-64		
Term	Estimate	CI-L	CI-U	Estimate	CI-L	CI-U
intercept	2.51	1.85	3.30	1.78	0.27	3.31
$\log_2$(trial)	0.09	-0.14	0.31	0.28	-0.01	0.58
HT	-0.30	-0.67	0.06	0.10	-0.18	0.37

Table 7

House type: duration regression parameter estimates. Reported numbers in log_2 seconds.

	Trials 1-16			Trials 17-64		
Term	Estimate	CI-L	CI-U	Estimate	CI-L	CI-U
intercept	4.29	4.14	4.44	4.58	4.26	4.90
$\log_2$(trial)	-0.13	-0.17	-0.09	-0.19	-0.24	-0.13
HT	0.32	0.26	0.38	0.05	0.02	0.09

Table 8

House type accounting for tutorial house type: accuracy regression parameter estimates. Reported numbers in logits.

	Trials 1-16			Trials 17-64		
Term	Estimate	CI-L	CI-U	Estimate	CI-L	CI-U
intercept	2.71	1.95	3.59	1.85	0.34	3.41
$\log_2$(trial)	0.10	-0.13	0.32	0.29	0.00	0.58
HT	-0.40	-0.95	0.13	0.02	-0.37	0.41
C	-0.42	-1.22	0.35	-0.22	-0.88	0.42
HT $\times$ C	0.18	-0.55	0.92	0.15	-0.40	0.70

Table 9

House type accounting for tutorial house type: duration regression parameter estimates. Reported numbers in log_2 seconds.

	Trials 1-16			Trials 17-64		
Term	Estimate	CI-L	CI-U	Estimate	CI-L	CI-U
intercept	4.32	4.12	4.52	4.67	4.32	5.02
$\log_2$(trial)	-0.13	-0.17	0.-0.09	-0.19	-0.24	-0.13
HT	0.33	0.24	0.42	0.04	-0.01	0.09
C	-0.05	-0.32	0.22	-0.20	-0.47	0.06
HT $\times$ C	-0.02	-0.15	0.10	0.03	-0.04	0.09

Table 10

Test phase regression credible intervals with and without random slopes.

		Random	Trials 1-16			Trials 17-64		
DV	Term	Slope	Estimate	CI-L	CI-U	Estimate	CI-L	CI-U
Acc.	DS	Yes	-0.10	-0.46	0.26	0.01	-0.24	0.28
Acc.	DS	No	-0.10	-0.45	0.25	0.02	-0.25	0.29
Acc.	HT	Yes	-0.30	-0.67	0.06	0.10	-0.18	0.37
Acc.	HT	No	-0.30	-0.65	0.05	0.09	-0.18	0.36
Acc.	GP	Yes	0.16	-0.25	0.56	-0.31	-0.65	0.01
Acc.	GP	No	0.17	-0.25	0.57	-0.31	-0.65	0.01
RT	DS	Yes	-0.02	-0.09	0.04	0.00	-0.03	0.03
RT	DS	No	-0.02	-0.09	0.04	0.00	-0.04	0.03
RT	HT	Yes	0.32	0.26	0.38	0.05	0.02	0.09
RT	HT	No	0.32	0.26	0.39	0.05	0.02	0.09
RT	GP	Yes	0.11	0.04	0.19	0.09	0.05	0.13
RT	GP	No	0.12	0.04	0.19	0.09	0.05	0.13

DS: digit set; HT: house type; GP: goal cell position; CI-L and CI-H: lower and upper bounds of the 95% credible interval. Accuracy (Acc.) coefficients are presented in logits and response time (RT) measure in coefficients are presented in log_2*(seconds).*

Table 11

Years of education regression parameter and R^2 estimates

Term	Estimate	CI-L	CI-U
intercept	36.24	20.67	51.82
education	1.45	0.43	2.47
R^2	0.032	0.002	0.08

Table 12

Math courses regression parameter and R^2 estimates

Term	Estimate	CI-L	CI-U
intercept	38.73	32.61	44.70
alg	9.65	2.88	16.67
geom	9.88	3.73	15.88
trig	3.14	-2.53	8.83
sv_calc	4.57	-2.2.52	11.62
mv_calc	-1.11	-9.22	6.88
linalg	0.50	-5.83	6.85
pr_stat	2.64	-2.21	7.41
disc	6.38	-3.08	15.79
logic	-1.24	-8.12	5.63
R^2	0.21	0.13	0.28

Table 13

Algebra and geometry courses regression parameter and R^2 estimates

Term	Estimate	CI-L	CI-U
intercept	40.78	35.05	46.57
alg	9.07	2.03	15.82
geom	12.69	6.96	18.43
R^2	0.15	0.08	0.22

Table 14

Years of education, algebra course, and geometry course regression parameter and R^2 estimates

Term	Estimate	CI-L	CI-U
intercept	25.66	10.34	40.85
education	1.04	0.06	2.00
alg	9.57	2.73	16.43
geom	11.48	5.62	17.16
R^2	0.17	0.10	0.24

Table 15

Years of education and math courses regression parameter and R^2 estimates

Term	Estimate	CI-L	CI-U
intercept	29.84	14.45	45.51
education	0.63	-0.38	1.62
alg	9.96	3.07	16.93
geom	9.38	3.63	15.26
trig	2.56	-3.09	8.38
sv_calc	4.74	-2.07	11.68
mv_calc	-1.41	-9.22	6.54
linalg	0.43	-6.08	6.87
pr_stat	2.13	-2.73	7.02
disc	6.14	-2.87	15.30
logic	-1.69	-8.58	5.15
R^2	0.21	0.14	0.29

Table 16

Questionnaire multiple choice responses rates and differences between persistent solvers and PD guessers

Measure	Mean PS	Mean PDG	Mean Diff	Diff CI-L	Diff CI-U
Attention Check	90.08%	82.54%	7.54%	1.68%	13.55%
Solved Puzzle	95.24%	50.00%	45.24%	32.75%	55.98%
Puzzle Confidence	96.43%	55.95%	40.48%	28.60%	51.18%
Noticed	80.95%	58.33%	22.62%	8.83%	35.78%
Checked Candidate	75.00%	45.90%	30.10%	12.24%	45.54%
Checked House	72.55%	36.36%	36.19%	12.33%	56.94%

Figure 11
Diagnostic Puzzle

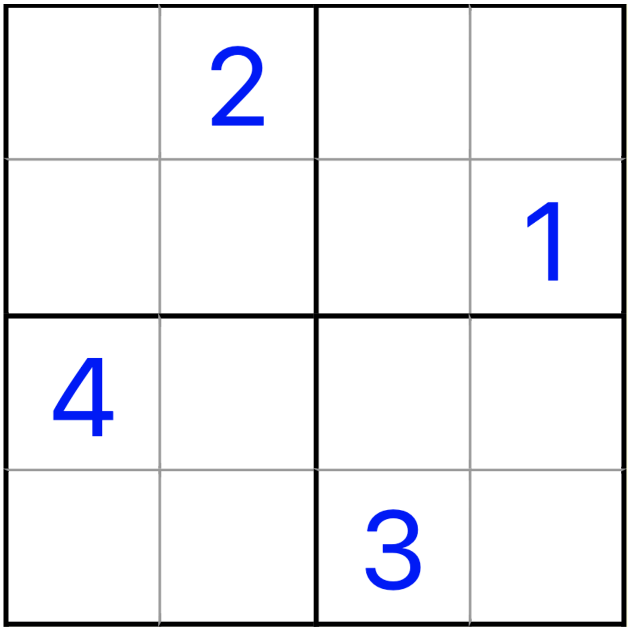

Figure 12

Posterior samples of feature coefficients for accuracy models.

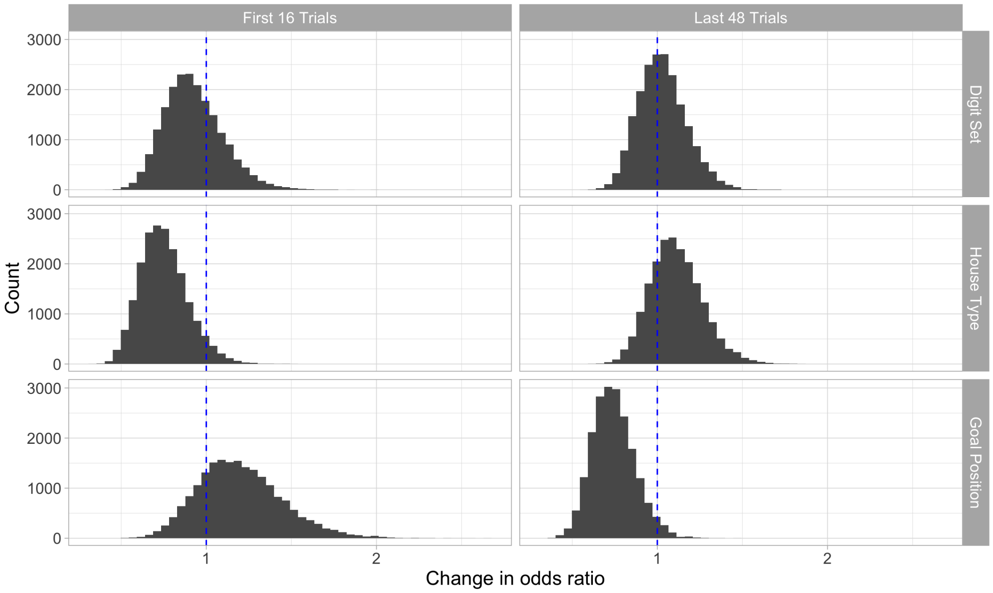

Figure 13

Posterior samples of feature coefficients for response time models.

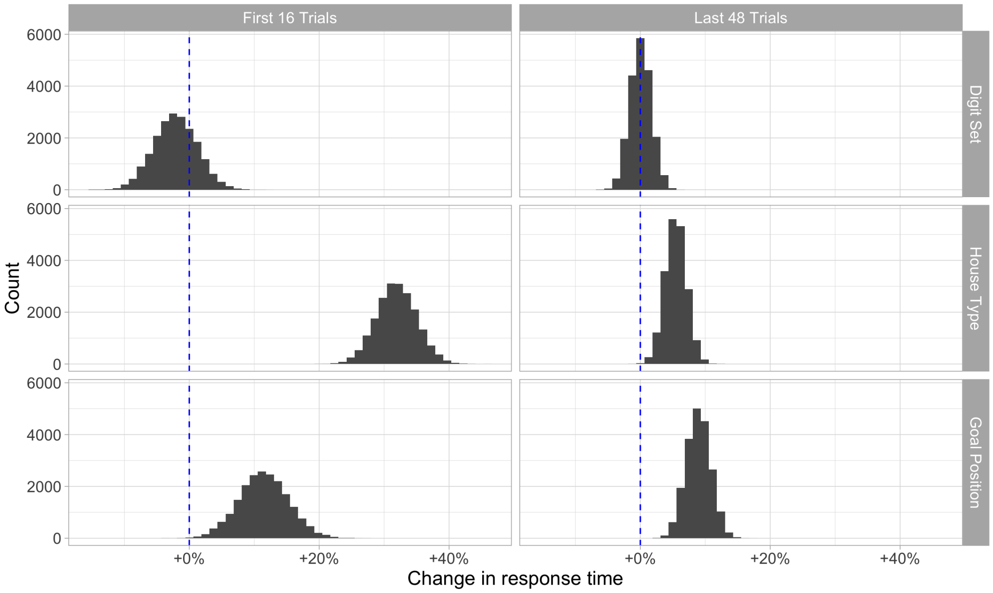

Figure 14

Quantitative responses by group. Total number of responses at the bottom of each bar for each question and group. Each participant was asked 3 attention check questions.

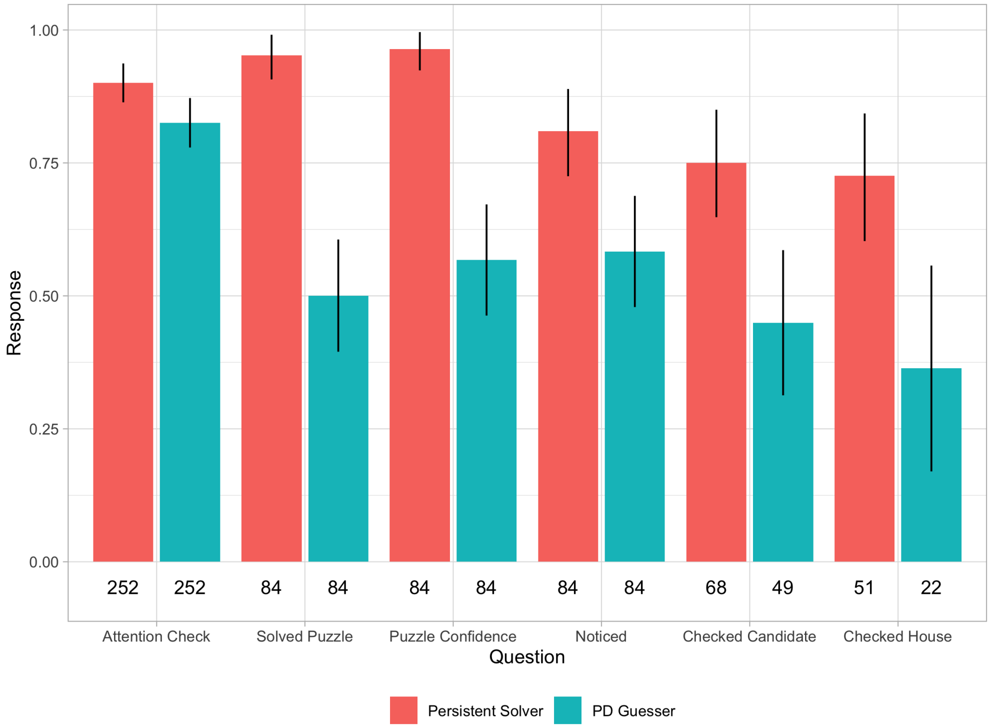

Figure 15

Ratings of self-reported strategies between persistent solvers and PD guessers. 'Other' category was never used by either rater.

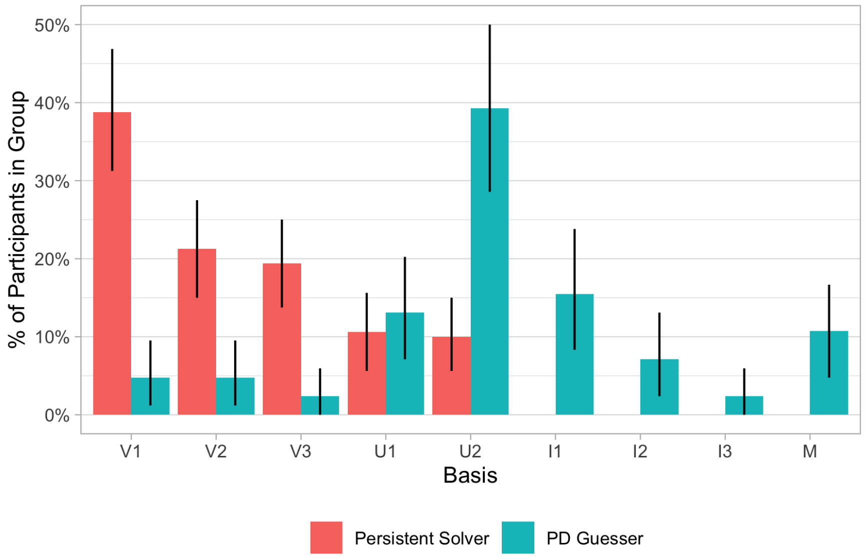

Figure 16

Left: A hidden single puzzle in which the shortcut variant of option V1 would produce the correct answer. Right: A hidden single puzzle in which the shortcut variant of option V1 would not produce the correct answer.

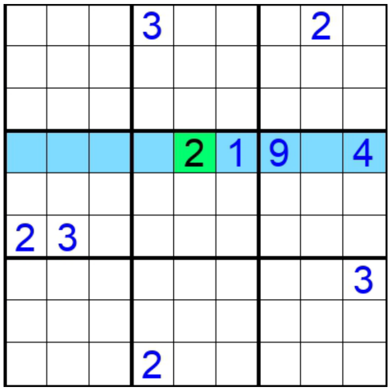

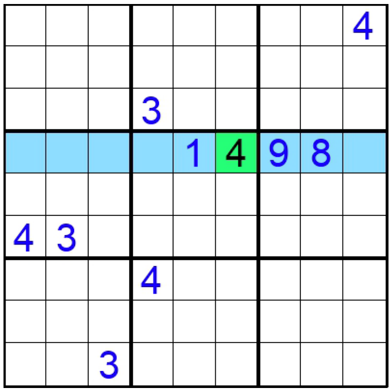

Figure 17

Prevalent Digit classifications.

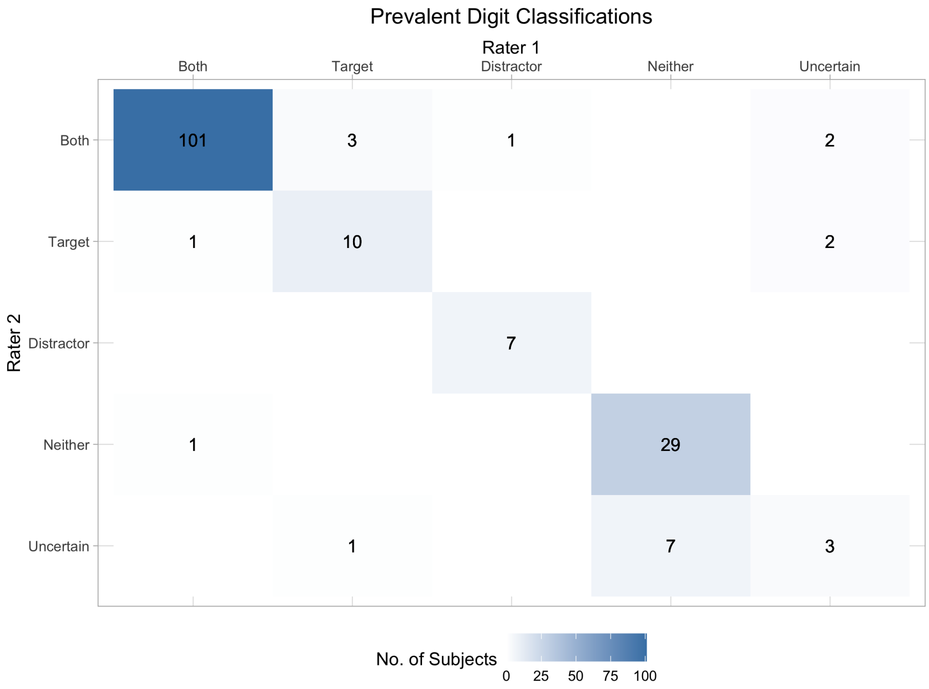

Figure 18

Superclass ratings of basis for choice by each rater. Only first choices shown.

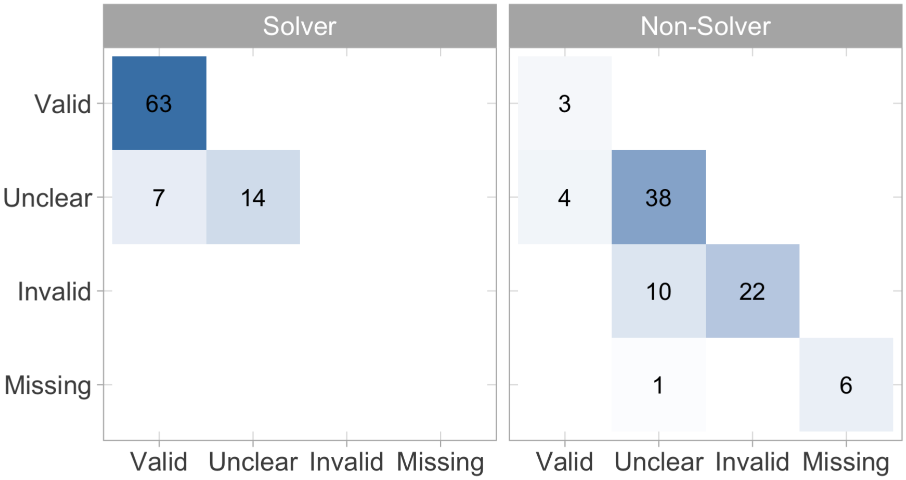

Figure 19

Subclass ratings of basis for choice by each rater. Only first choices shown.

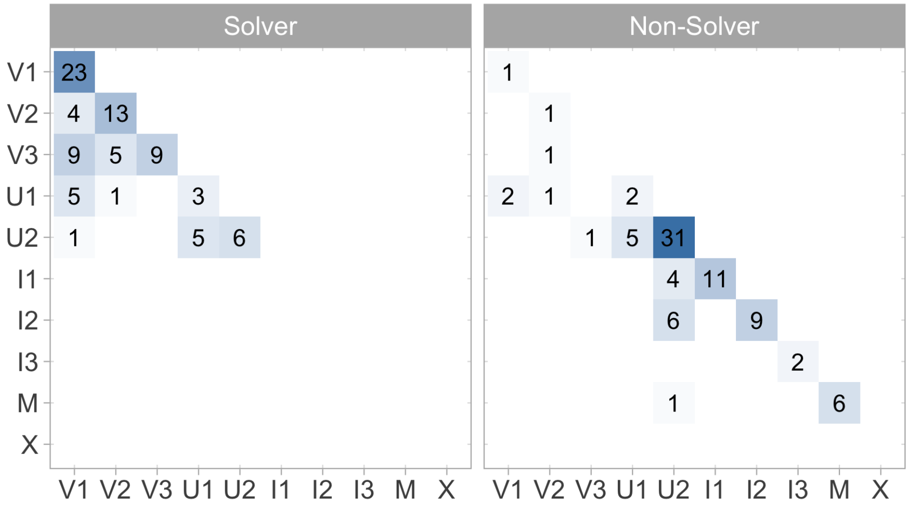

Figure 20

Correlation matrix of participants' task performance and education variables. 'Solver' is a binary variable of whether they had been identified as solvers or not.

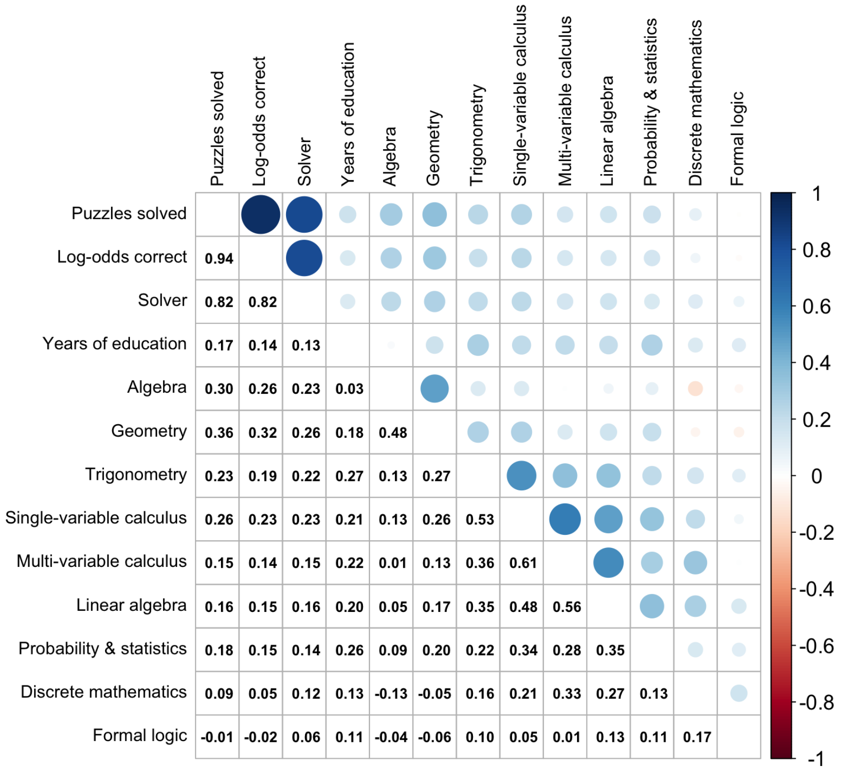

Figure 21

Replication results of Palm et al. on full 9x9 Sudoku puzzles. Results show averages across 10 different model instances.

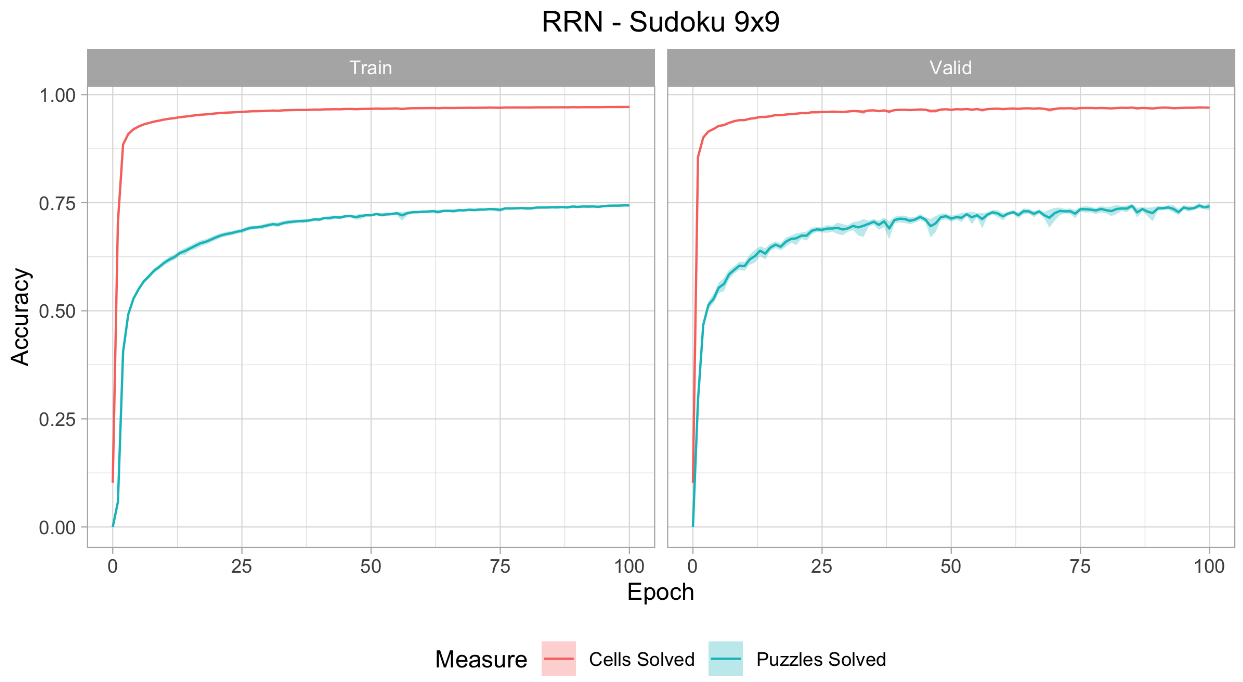

Figure 22

(a-b) Recurrent relational network's (RRN) and Digit-invariant RRN's (DRRN) validation accuracies on held-out puzzles with the same features as training samples. The x-axis represents total number of training puzzles presented to the model and the y-axis represents cumulative maximum accuracy during training. 10 models were trained for each training set size. Best and Worst lines indicate the highest and lowest cumulative maximum accuracy among the 10 model instances respectively. For the DRNN, training set size made very little difference, with best and worst curves for most set sizes falling on top of each other in the figure. (c) Test set accuracy by feature for human solvers, the RRN, and the DRRN. Error bars indicate 95% highest density intervals over model runs or human participants. Note the failure of the RNN to perform well on held-out target digits, indicated by the red dot for that condition in the middle panel of the figure.

(a) *RRN - Validation Set Accuracy*

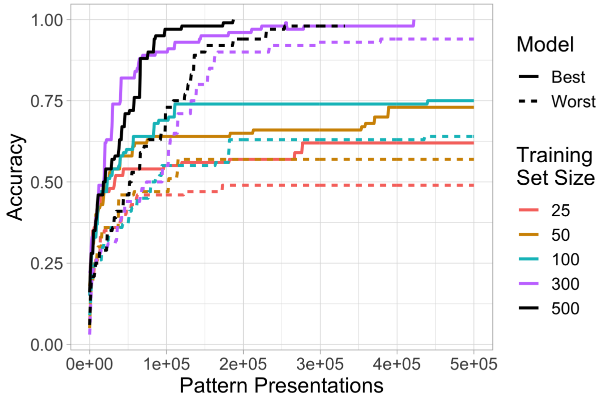

(b) *DRRN - Validation Set Accuracy*

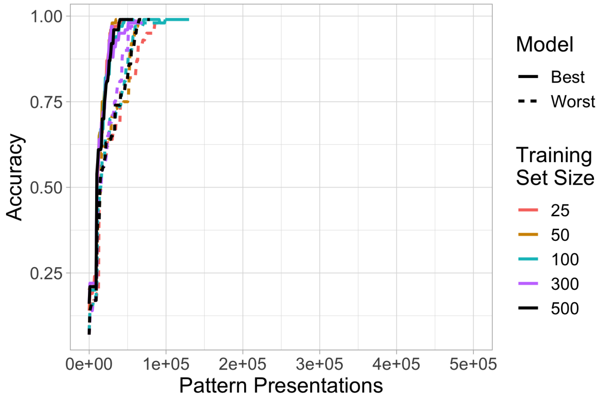

(c) *Test Set Accuracy*

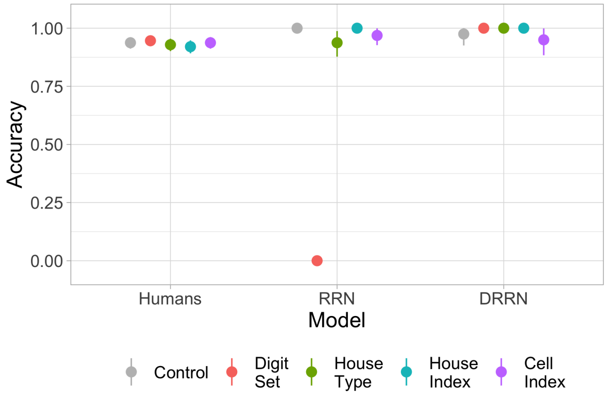

Figure 23

Accuracy of relational networks on training and held-out validation (puzzles sharing features as training set) sets. Results show average performance across 10 different model instances trained on 10 different datasets. The same 10 datasets were used to train both RRNs and DRRNs.

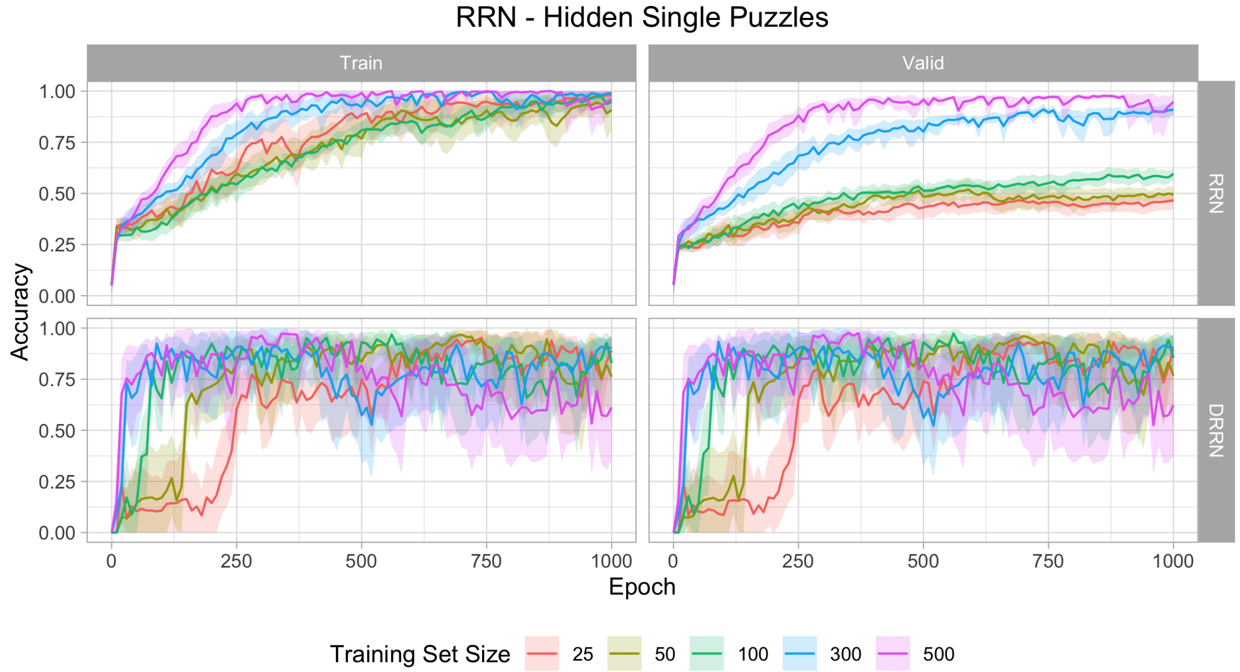

Figure 24

Accuracy of relational networks on held-out test set with systematically varied features (marginalizing over others), evaluated on models trained with 500 puzzles selected on epochs with highest validation accuracies. Results show average performance across 10 different model instances. Error bars indicate 95% highest density intervals.

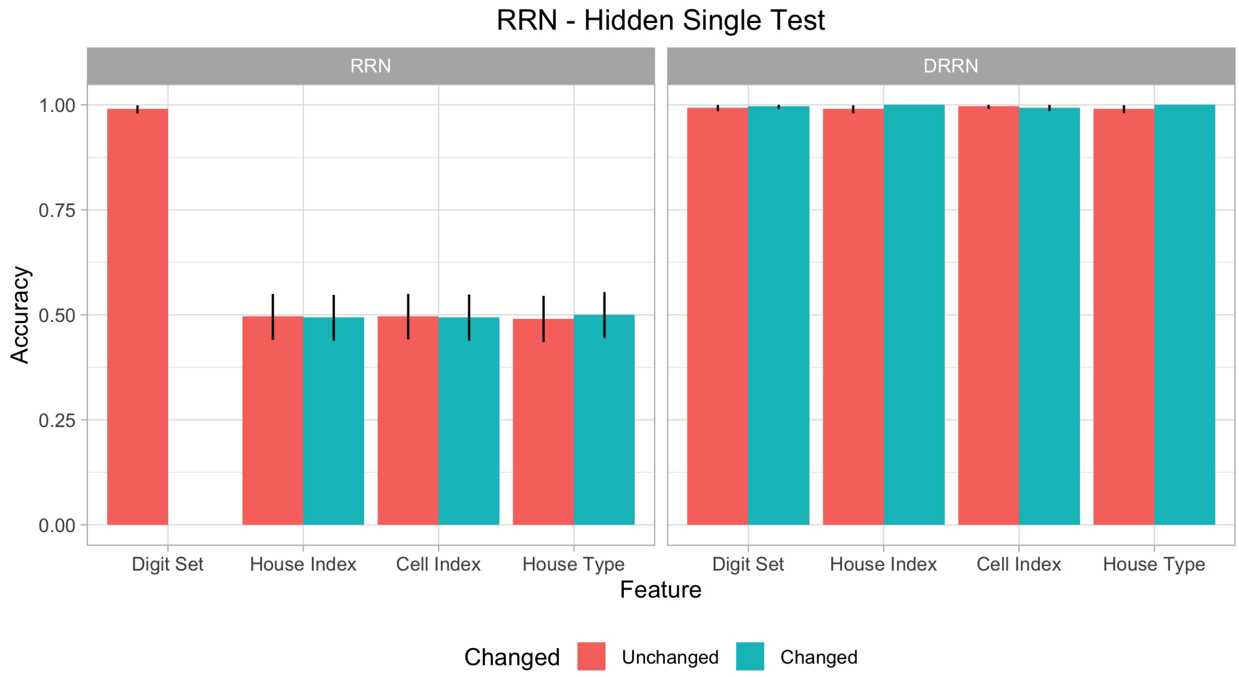

Figure 25

Accuracy of DRRN on training and held-out validation sets. Each plot shows a different instance of the model trained on different sets of 500 training puzzles.

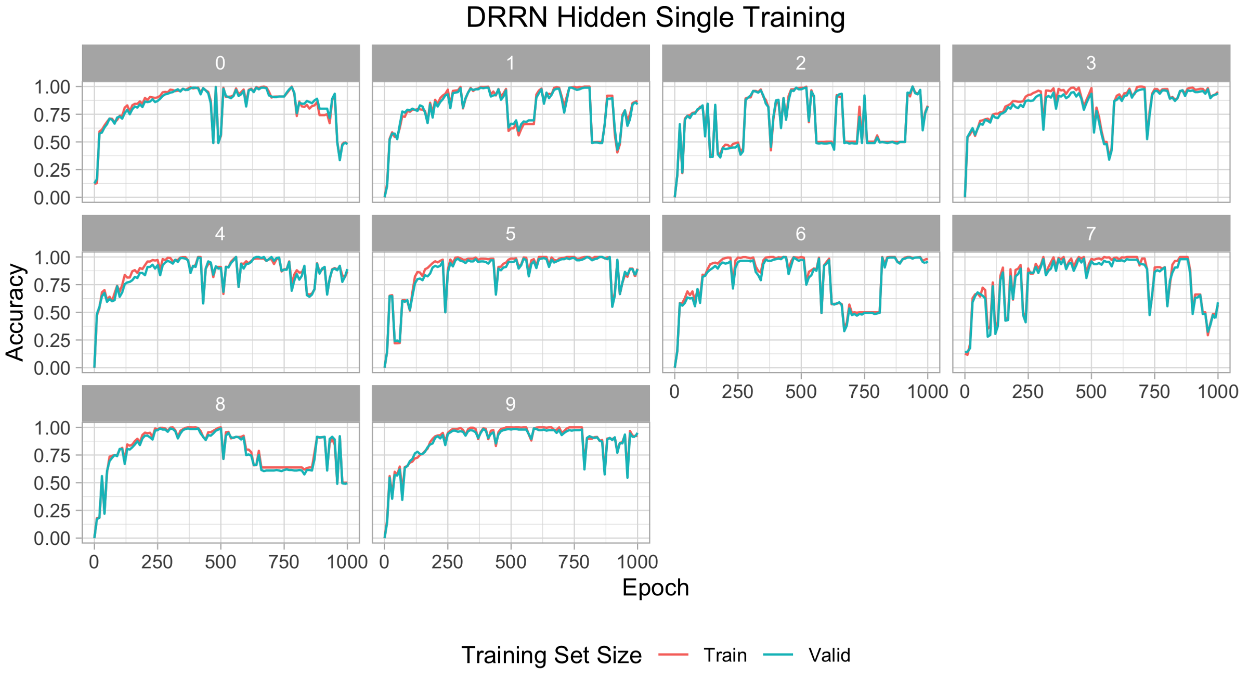

Supplement: Supplementary file 1 [file opmi-08-148-s001.pdf]
